# Microbial niche differentiation and agronomic performance of diseased *Capsicum annuum*

**DOI:** 10.3389/fmicb.2025.1576486

**Published:** 2025-09-03

**Authors:** Zhiqi Yang, Yankun Wang

**Affiliations:** ^1^College of Agriculture, Fujian Agriculture and Forestry University, Fuzhou, Fujian, China; ^2^College of Bee Science and Biomedicine, Fujian Agriculture and Forestry University, Fuzhou, Fujian, China

**Keywords:** long line pepper, microbial community, pathogen, niche differentiation, agronomic performance

## Abstract

**Introduction:**

Plant–microbial interactions shape the plant microbiome, leading to niche differentiation in microbial communities. The dynamic variation in beneficial and phytopathogenic microbes from different niches (including the roots, stems, leaves, and rhizosphere soil (RS) of plants) is poorly understood.

**Methods:**

High-throughput sequencing was performed to explore the shifts in microbial community composition in different niches of diseased and healthy long line peppers (LLPs, *Capsicum annuum* L.). Correlations between microbial community composition and agronomic performance were analyzed to speculate the presence of potential pathogens and beneficial microbes in different niches and their effects on LLPs.

**Results:**

The relative abundance of microbial communities in the LLP different niches was dynamic. Some microbes exhibited significantly negative effects on the LLP growth and fitness, including the genera bacterial *Pseudomonas*, *Pectobacterium* and fungal *Fusarium*, *Alternaria*, *Xepicula*, *Mrakia*, and *Verrucoconiothyrium*. Two pepper-wilt fungi *F. proliferatum* and *F. oxysporum* were identified according to Koch’s rule, validating the study’s conclusions. The pepper disease reduced plant fresh weight by 72% and increased *Fusarium* abundance by 2-fold, additionally, LLP plant height, concentrations of leaf chlorophyll a, fruit vitamin C and fresh weight were significantly decreased contrast to healthy plants. Certainly, potential beneficial microbes (e.g., the *Priestia*, *Occallatibacter*, and *Enterobacter* bacteria as well as the *Sporobolomyces*, *Hannaella*, *Verticillium*, *Bisifusarium*, and *Vishniacozyma* fungi) significantly promoted some agronomic parameters of LLPs.

**Conclusion:**

These finding suggested that various pathogens might be associated with pepper disease symptoms. This study lays a foundation for isolations, identifications, experimental validation of phytopathogens and beneficial microbes.

## 1 Introduction

The plant microbiome consists of microbial communities that reside within and on the surface of plants (Xu et al., 2022; Aziz et al., 2022; Turner et al., 2013). The beneficial microbes inside plants have coevolved with their hosts for a long time and established mutualistic interactions with the host plants (Utami et al., 2022; Ahlawat et al., 2022; Chen et al., 2020). These microbes take part in many life processes of plants, for example, the secretion of hormones (e.g., production of the auxin indoleacetic acid (IAA) that improves plant growth); promotion of plant nutrient (such as nitrogen and phosphorus) uptake; regulation of the plant immune system; and increased resistance to pathogens (Bolívar-Anillo et al., 2020; Tripathi et al., 2022). However, phytopathogens, which infect roots, stems, leaves, and fruits, are harmful to the growth and health of plants and ultimately lead to a decrease in crop productivity and economic losses (De Rossi et al., 2021). For instance, blast, rust, smut, and blotch diseases of wheat (*Triticum aestivum* L.) are caused by fungal phytopathogens (Figueroa et al., 2018), while fire blight of apple (*Malus pumila* Mill.) and black rot and bacterial speck disease of tomato (*Solanum lycopersicum* L.) are caused by bacterial phytopathogens and can lead to considerable crop losses (Savary et al., 2019). Phytopathogenic diseases cause billions of dollars in economic losses annually (Figueroa et al., 2018). Additionally, pepper disease development, microbial community composition and diversity are influenced by environmental characteristics such as soil properties, soil enzymatic activities, the weather and others (Shen et al., 2023; Cui et al., 2020; Dai et al., 2019). Therefore, characterizing potential beneficial microbes and pathogens for their application to crop production is important for further protecting crops from attack by phytopathogens.

Phytopathogens are recognized as pathogenic microbes which can be isolated from diseased parts of plants; these isolated microbes will then infect host plants and cause typical symptoms, confirming their role using Koch’s postulates. Subsequently, the morphological characteristics of the cultured colonies are one of the bases of classification, and the isolated pathogens are identified via high-throughput sequencing and alignment with published sequences from databases (Han et al., 2020; Li et al., 2021; Mancini et al., 2016). For example, plant root diseases can be caused by the fungal pathogens *Fusarium oxysporum* and *Rhizoctonia solani*, which can be cultured on agar media plates and identified by alignment of the rRNA internal transcribed spacer (ITS) region (Li et al., 2021; Mancini et al., 2016; Woodhall et al., 2022). Bacterial spots on tomato and pepper plants are caused by four species from the genus *Xanthomona*s, namely *X. vesicatoria*, *X. euvesicatoria*, *X. gardneri*, and *X. perforans*, which can be cultured on solid agar media and identified using 16S rRNA sequence alignment (Jibrin et al., 2018; Timilsina et al., 2015; Richard et al., 2017). However, control strategies for crop diseases commonly do not meet the expectations of agricultural productivity (Figueroa et al., 2018) because only a small subset of pathogen communities can be isolated from diseased plants, cultured, and then successfully identified (Potnis et al., 2011). Gene sequencing (16S rRNA for bacteria, ITS for fungi, metagenomics) is a culture-independent technique that can be used to evaluate microbial diversity and the composition of pathogen communities in host plants. Previous researchers have focused mainly on investigating diseased plant organs or plants (Li et al., 2021; Woodhall et al., 2022; Jibrin et al., 2018; Richard et al., 2017). Based on the correlations between RS, endophytic microbial communities in tissues, and agronomic performance, few phytopathogens have been identified by gene sequencing. Although some endophytic pathogens have been isolated from diseased peppers, their microbiome-wide roles remain unclear.

Long line pepper (LLP, *Capsicum annuum* L.) is an annual vegetable crop of the Solanaceae family. In China, Guizhou Province had the largest area (0.2 million hm^2^) of dried hot pepper plantations in 2022, most of the varieties being pod and LLPs, making pepper production one of the major crop types of Guizhou Province (Qiao et al., 2023). In recent years, with the rapid development of pepper plantations, pepper various diseases have also become more common in pepper-growing areas, such diseases being widely distributed and harmful, becoming the main obstacle to the sustainable, healthy, and efficient development of the pepper industry (Bhagat et al., 2023; Bezabih et al., 2023; Liu and Wang, 2024; Liu et al., 2022). *Fusarium* wilt is one of the pepper common diseases, the incidence ratio has reached to 30%, frequent or yearly cultivation leads to a higher incidence of the wilt disease (Zhang et al., 2022; Zhang et al., 2021). However, most of the previous studies on hot pepper disease have focused on the symptoms of infected plants and the isolation and identification of pathogens (Tian et al., 2023; Wang et al., 2020). The dynamic fluctuations in microbial communities, especially of pathogens, in diseased LLPs are poorly understood.

In the present study, we hypothesize that specific bacterial and fungal communities significantly influence pepper plant fitness and disease resistance. To test these hypotheses, we investigated how the RS and tissue endophytic microbes of LLP plants vary under wilt disease stress and tested the corresponding agronomic parameters of LLP plants and fruits. To address the topics mentioned above, we collected samples of RS and tissues from diseased and healthy LLP plants from the same field in Zhongcheng Village, Rongjiang County, Guizhou Province, in Southwest China. A comparison was performed of the composition and relative abundance of microbial communities and the corresponding agronomic performance of LLPs in plants of the two health states (healthy and diseased). Microbial niche differentiation (RS, root, stem, and leaf distributions) was determined, and the correlations between microbe frequencies and plant agronomic performance were analyzed. Our study aimed to address: (1) Compositions and differences in microbial communities from RS and tissues (roots, stems, and leaves) of diseased and healthy LLP plants; (2) comparisons of agronomic performances in diseased and healthy plants of LLP, and identifying microbial taxa with disease symptoms and agronomic decline; and (3) the existent possibility, and fluctuation of potentially pathogenic microbes, and candidate pathogenic identifications and validation. We expected to understand that pathogens were harmful to the host pepper, and potential beneficial microbial communities were conducive to pepper growth and fitness. More importantly, this study provides insights into the existent possibility of various phytopathogens by the exploitation of LLP microbial communities, and lays a foundation for isolations, identifications, experimental validation of others phytopathogens.

## 2 Materials and methods

### 2.1 Site description and sample collection

To verify our hypothesis, pepper plants with typical wilt symptoms were selected after consulting experts and reviewing the relative literatures. Hot pepper field located in Zhongcheng Village, a suburb of Rongjiang County, Guizhou Province, in China (25°99′08″ N; 108°55′68″ E; altitude of 276 m above sea level), which has a flat terrain and is surrounded by mountains. This area experiences a hot and humid climate, with abundant rainfall, and an annual frost-free period of more than 340 days. The average annual temperature is approximately 19 °C, the average annual precipitation is 1,249 mm, the average annual evaporation is 860 mm, the average annual relative humidity is approximately 80%, the average annual sunshine duration is approximately 1,152 h, the annual accumulated temperature is more than 6,500 °C. The soil is calcareous yellow soil, and the environment and air quality are suitable for growing vegetables, this vegetable field has long rotation plantation history of hot peppers and Chinese cabbage.

The RS and plant organ samples were collected from diseased and healthy LLPs in a same field. The cultivar (King liner pepper) is thin and long line cayenne pepper, which were subjected to same fertilization and water management. Following local fertilization practice, the LLP crop received 90 kg P_2_O_5_ ha^–1^, 125 kg N ha^–1^, and 115 kg k_2_O ha^–1^ in total fertilizers, a half of the fertilizers was decomposed pig mature as basic fertilization, another half was chemical fertilizers applied after manuring (Yin et al., 2022). Nine plants of similar sizes, stage of development, and disease symptoms (yellower leaves, thinner fruit and more red fruits) were randomly dug up using a shovel, all the roots with soil of a plant were pulled up, each taken at least 2 m from one another, making up three diseased replicates, with three plants per replicate; similarly, nine healthy plants of the same size and development were randomly sampled representing three healthy replicates. After the loose soil was shaken off, the soil adhering tightly to the roots, which was the RS of the plants, was collected into a sterile bag (Jin et al., 2018). All the soil and plant samples were placed in sterile plastic bags, which were subsequently placed in ice boxes and transported to the laboratory for sample preparation.

### 2.2 Sample preparation

After the fresh weight and height of the LLP plants in each health state were measured, the roots, stems, leaves, and fruits were separated into different organs and subsequently washed with running water; after the surface water was dried, the different organ samples were stored at 4 °C for subsequent analysis. Approximately 3 g of leaves, 2 g of roots, or 3.5 g of stems were surface sterilized with 75% (v/v) ethanol for 1 min, rinsed three times using sterile water, surface sterilized with 1% (v/v) sodium hypochlorite for 30 s, and finally rinsed eight times using sterile water (Wang et al., 2023). The fine root and plant residues were removed from the soil on the ultra-clean table. All the sterilized samples (3 organs × 3 replicates × 2 health states) and 5 g of RS (3 replicates × 2 health states) were put into separate sterile plastic bags and stored at −80 °C for DNA sequencing of bacteria and fungi.

### 2.3 Determination of soil physical and chemical properties

The physical and chemical properties of the soil were measured using a Soil Nutrient Speedometer (Model: LD-TYC, Shandong Laiende Intelligent Technology Co., Ltd., Weifang City, China) except for pH (Yang et al., 2025a). A total of 10 g of the soil sample was weighed and put it into a 50 mL beaker, then 25 mL of distilled water was added to fully mix the soil and water to form a soil suspension. Stirring the soil suspension to make it uniform to obtain the solution, pH was measured using a glass electrode (PHS-3E, Shanghai Lei-Magnetic Instrument Co., Ltd. Shanghai, China). The concentrations of soil organic matter (OM, g kg^–1^) were determined, specifically, 4 g of air-dry soil sample was weighed into the extraction bottle, and then 20 mL of organic matter extraction agent of soil organic matter kit was added. After shaking for 5 min, filter. The filtrate can be used to determine soil OM. Available nitrogen (AN, NH_4_^+^-N, mg kg^–1^), available phosphorus (AP, mg kg^–1^), and available potassium (AK, mg kg^–1^) were analyzed using Soil Nutrient Test kits (which integrated with the Soil Nutrient Speedometer in a box were purchased from Shandong Laiende Intelligent Technology Co., Ltd.) according to the manufacturer’s protocols. Specific process: 1.0 g of air-dry soil sample was weighed, 20 mL of the extraction solution and 0.1 g of soil decolorizer of soil kit were put into the bottle for extraction, and then shake violently for 3 min, filter. The filtrate is the soil available nutrient solution which could be used to determine soil AN, AP and AK.

Soil enzyme activities were measured according to the methods of Guan (1986). Phosphatase activity (PHA) assay used 4-NPP (4-nitrophenyl phosphate disodium salt hexahydrate) colorimetry, with air-dried soil (1 g) being mixed with pH 6.5 buffer solution containing 2.1 g L^–1^ 4-NPP and incubated at 37 °C for 24 h, with the *p*-nitrophenol (*p*-Nit) produced being determined by absorbance at 420 nm by spectrophotometry (759S, Lengguang Technology Co., Ltd., Shanghai, China) to measure soil PHA (mg *p*-Nit g^–1^ 24 h^–1^). Sucrase activity (SUA) was assayed using sodium thiosulfate titration, the soil (5 g) being mixed with pH 5.5 buffer solution containing saccharose; after incubation at 37 °C for 24 h, glucose produced was titrated to show soil SUA (mg glucose g^–1^ 24 h^–1^). Urease activity (URA) was assayed using phenol-sodium hypochlorite colorimetry; 5 g soil were mixed with pH 6.7 buffer solution containing urea, and incubated at 37 °C for 24 h, with NH_4_^+^-N formed being measured by absorbance at 578 nm to determine soil URA (mg NH_4_^+^-N g^–1^ 24 h^–1^).

### 2.4 Measurement of plant growth and physiological parameters

The plant height was measured from the stem base to the top of the LLP plant, and the fresh weight of the whole plant with fruits was determined. Physiological parameters were determined according to the methods of Gao (2006). Chlorophyll a and b concentrations were measured using colorimetry in 80% (v/v) acetone solution. The leaf sample (approximately 0.25 g) was cut into pieces and ground with CaCO_3_, quartz sand, and acetone solution in a mortar. After the tissue being ground were fully decolorized, the acetone solution was filtered into a 25 mL volumetric flask and subsequently adjusted to the constant volume. The chlorophyll a and b concentrations were determined by absorbance at 645 and 663 nm using spectrophotometry. Malondialdehyde (MDA) concentration was analyzed by thiobarbituric acid colorimetry, with approximately 0.3 g leaf pieces being ground with quartz sand and phosphate butter solution (pH, 7.8) until homogenization was achieved, at which point the homogenate was transferred to a tube and 5 mL 0.5% thiobarbituric solution was added. After heating for 10 min in a boiling water bath, followed by rapid cooling, the mixture was adjusted to 10 mL volume and centrifuged at 3, 000 × *g* for 15 min. Absorbance of the supernatant was measured at 450, 532, and 600 nm by spectrophotometry to determine the MDA concentration. Superoxide dismutase (SOD) activity was determined by the nitro blue tetrazolium (NBT) photoreduction method. Leaf pieces (0.5 g) were ground in an ice bath and then transferred to a 10 mL volumetric flask and mixed to a constant volume. After centrifugation at 3, 000 × *g* for 15 min, the supernatant (0.1 mL) was mixed with phosphate butter (50 mmol L^–1^, pH 7.8), methionine (130 mmol L^–1^), NBT (750 μmol L^–1^), EDTA-Na_2_ solution (100 μmol L^–1^), and riboflavin (20 μmol L^–1^). After the reaction, which proceeded under the light of a 4, 000 lx purple lamp for 15–20 min, the reaction was stopped by turning off the lamp and the absorbance of the reaction mixture was finally measured at 560 nm using spectrophotometry to determine SOD activity. Peroxidase (POD) activity was measured using the guaiacol method. Approximately 0.5 g of leaf pieces were ground in pH 7.8 buffer solution. After centrifugation at 3, 000 × *g* for 15 min, the supernatant was transferred to a 25 mL volumetric flask and added deionized water to 25 mL, which was crude enzyme extract. Afterward, 0.5 mL of the enzyme extract was mixed with 1.0 mL 0.1% guaiacol solution. After the reaction, the absorbance at 470 nm was determined using spectrophotometry to evaluate POD activity (Hernández-Esquivel et al., 2020). Proline concentration was measured by the ninhydrin colorimetry method, with 0.3 g fresh leaf pieces being ground into a homogenate, which was transferred to a tube with a plug, then 5 mL 3% sulfosalicylic acid solution was added and the tube was covered with the plug. After extraction in a boil bath for 15 min, the extracted solution was filtered, and 1.5 mL deionized water, 2 mL glacial acetic acid, and 2 mL acid ninhydrin solution were added to 0.5 mL of the filtrate in a tube. After the tube was shaken, it was placed in an ice bath for 30 min, then 5 mL methylbenzene was added and mixed well, placed in the dark for 2–3 h, and the proline concentration was determined by measuring the absorbance at 520 nm by spectrophotometry.

### 2.5 Evaluation of fresh fruit mass and quality

The fresh weight of five ripe fruits was weighed to calculate the mean fresh mass. The quality of LLP fruit was evaluated by measuring the following parameters (Li and Zhang, 2016), namely the concentrations of vitamin C (Vc), soluble sugar, and soluble protein in fresh fruits. Vc concentration was tested by iodometry. A 2.5 g subsample of fresh fruit was ground and put into a 250 mL Erlenmeyer flask, mixed with deionized water, boiled, and subsequently cooled. Then, 10 mL 2 mol L^–1^ acetic acid and 5 mL 0.2% (w/v) starch solution were added, respectively, and the solution was immediately titrated with I_2_ standard solution until a stable light blue color developed and did not fade within 30 s. The volume of I_2_ standard solution used in the titration was recorded and the Vc concentration was calculated. The concentration of soluble sugar was determined *via* the anthrone colorimetric method. A 0.3 g fruit subsample was ground with 4 mL 80% (v/v) ethanol, followed by agitation of the homogenate in an 80 °C water bath for 30 min, and centrifugation at 2, 775 × *g* for 10 min. The supernatant was transferred to a 10 mL graduated tube and 0.5 g activated carbon were added for decolorization at 80 °C, the volume adjusted to 10 mL and filtered, with the filtrate being used to determine the concentration of soluble sugars. The soluble protein concentration was measured by the Coomassie Brilliant blue colorimetric method. After 0.2 g fruit was ground, the homogenate was transferred to a 10 mL volumetric flask and adjusted to 10 mL with deionized water, then 2 mL of this diluted homogenate solution was centrifuged at 5, 000 × *g* for 10 min. An aliquot (0.1 mL) of the supernatant was mixed with 0.9 mL deionized water and 5 mL Coomassie Brilliant blue G-250 reagent; after allowing color to develop for 2 min, the soluble protein concentration was determined from the absorbance at 595 nm by spectrophotometry.

### 2.6 Microbial DNA extraction, amplification, and sequencing

Subsamples of all 24 soil and plant samples, including 5 g of RS, 1.5 g of roots, 3.5 g of stems, and 3 g of leaves, were frozen and immediately sent to the laboratory in a dry ice box for microbial DNA extraction. The roots, stems, and leaves were sterilized with 75% alcohol three times and then rinsed with PBS (phosphoric acid buffer solution sterilized, pH 7.0) three times. Approximately 200–500 mg of the soil sample was put into a 2 mL sterile tube, 1 mL PBS was added, the mixture was mixed well, and centrifuged at 24, 200 × *g* for 3 min. The supernatant was discarded, and the liquid in the soil pellet was allowed to drain away. After all samples were individually ground with liquid nitrogen, the microbial DNA was extracted using an E.Z.N.A. Mag-Bind Soil DNA Kit (M5635-02, Omega Bio-Tek, Norcross, GA, United States), because of the wide application scope of the kit, following the manufacturer’s instructions (Niu et al., 2017; Yin et al., 2021). The quality of the extracted DNA was examined using 1% agarose gel electrophoresis (Supplementary Figure 1) and quantitated using a Qubit^®^ 4.0 fluorimeter (Q33238, Thermo Fisher Scientific, Waltham, MA, United States) (Supplementary Table 1). During the first amplification, the V3–V4 region of bacterial 16S rRNA was amplified with the universal primer pair 341F 5′-CCTACGGGNGGCWGCAG-3′ and 805R 5′-GACTACHVGGGTATCTAATCC-3′ (Zhang et al., 2019), the both primers were selected for V3–V4 amplification of 16S rRNA due to a high coverage rate in bacterial and could detect the diversity distribution of bacteria. The fungal internal transcribed spacer (ITS)1 region was amplified by the primer pair ITS1F 5′-CTTGGTCATTTAGAGGAAGTAA-3′ and ITS2R 5′-GCTGCGTTCTTCATCGATGC-3′, the ITS sequence is a small, highly variable gene region which is often used to identify a specific species, and these two fungal primers were selected for fungal ITS1 region amplification, a wider variety of species were gathered (Li et al., 2024; Liu et al., 2024). In the first amplification, A 30 μL reaction mixture, which contained polymerase (2 × Hieff^®^ Robust PCR Master Mix, 10105ESO3, Yeasen, Shanghai, China), primer pair, PCR products, and sterile deionized water (Supplementary Table 2), was amplified using a Polymerase Chain Reaction (PCR) instrument (Beijing Dongsheng Innovation Biotechnology Co., Ltd., Beijing, China) with the following reaction conditions: 94 °C, 3 min →→ (94 °C, 30 s → 45 °C, 20 s → 65 °C, 30 s) _5_ →→ (94 °C, 20 s → 55 °C, 20 s → 72 °C, 30 s) _20_ →→ 72 °C, 5 min →→10 °C, ∞. For the second amplification, Illumina bridge PCR compatible primers (Shanghai BoHu Biotechnology Co., Ltd., Shanghai, China) were used for the reaction system, including polymerase, adapter pair, the first amplification PCR products, and sterile deionized water (Supplementary Table 2), and the PCR conditions were 95 °C, 3 min →→ (94 °C, 20 s → 55 °C, 20 s → 72 °C, 30 s) _5_ →→ 72 °C, 5 min →→10 °C, ∞. Amplification results were presented in Supplementary Figures 2, 3. After qualification via 2% agarose gel electrophoresis, the purified amplicons were sequenced using the Illumina MiSeq PE300 platform (Illumina MISeq, San Diego, California, United States). All the DNA extraction, amplification, and sequencing steps were performed at Sangon Biotech Co., Ltd., Shanghai, China.

### 2.7 Bioinformatic analysis

The raw fungal and bacterial sequences were processed by the following methods. Briefly, the primer adapters of paired-end (PE) sequences were removed by Cutadapt (version 1.18) (Martin, 2011) and merged into a read using PEAR (version 0.9.8) according to the overlap of PE reads. The sample data were subsequently segmented from the spliced data according to the primer and barcode sequence of each sample, after which the direction of the sequence was corrected (Zhang et al., 2014). PRINSEQ (version 0.20.4) was applied to remove low*-*quality reads (those shorter than 20 bp) from the reads downstream, filter out N-containing sequences, and short sequences after quality control, and ultimately filter out low-complexity sequences (Schmieder and Edwards, 2011). Operational taxonomic units (OTUs) with a 97% sequence identity cut-off value were clustered by UPARSE (version 7.1), chimeric sequences were removed (Edgar, 2013) to obtain the OTU representative sequence, which was aligned using RDP Classifier (version, 2.12) for bacteria and USEARCH (version 11.0.667) for fungi, and sequences with 97% identity or greater were selected for the OTU data (Edgar, 2016). The taxonomic annotation of all sample OTU representative sequences was conducted using the SILVA database^1^ for bacteria (Quast et al., 2013; Xu et al., 2024) and the UNITE database^2^ for fungi (Altschul et al., 1997). After sequence annotation, non-16S sequences (e.g., plant mitochondria and chloroplasts) were removed (Feng et al., 2023). To reduce the natural noise and compare the differences among RS and organs LLP samples with different sequencing read count, R Package phyloseq (1.30.0) was applied to transform sample read count OTU data into the microbial relative abundances (McMurdie and Holmes, 2013), which were normalized by R package DESeq2 (1.26.0) (Anders and Huber, 2010; Knight et al., 2018). At last, the sample rarefaction, the microbial diversity, richness, and community composition of each sample was calculated at different classification levels.

### 2.8 Statistical analysis

Microsoft Excel 2016 (Microsoft Corp., Redmond, WA, United States) was used to calculate the soil physicochemical properties and enzymatic activities, plant growth and physiology parameters, and fresh fruit weight and quality, and to generate the bar charts. Via SPSS (version 23.0; SPSS Inc., Chicago, IL, United States), the normality and homogeneity of the variances had been tested using Shapiro-Wilk and Levene, respectively, these soil, plant and fruit data for diseased and healthy pepper plants were subjected to paired-sample *T*-test of variances. The differences between the two health states (diseased/healthy) were determined, and the significance level was *P* ≤ 0.05.

The alpha-diversity (ACE and Shannon indices) of the bacterial and fungal communities was analyzed by Mothur (version 1.43.0) based on OTU richness (Schloss et al., 2009), and box plots were generated via the R “ggplot2” package. The ACE (Abundance-based Coverage Estimator) index, proposed by Chao, serves as a metric for estimating the number of OTUs (richness) within a community; the Shannon index was used to estimate the bacterial and fungal diversity. The data for the microbial ACE and Shannon indexes were evaluated for normality and homogeneity used Shapiro-Wilk and Levene, these two alpha-diversity indexes were subjected to one-way analysis of variance (ANOVA) and the microbial niche differences were compared using Least Significant Difference (LSD). Principal coordinate analysis (PCoA) for bacterial and fungal beta-diversity was used to show the eigenvalue of the sample distance matrix (Bray–Curtis distance coefficients) calculated by the R “vegan” package (version 2.5-6), and the results were visualized using the R “ggplot2” package on the OmicShare cloud tool^3^ (Gao et al., 2019). And permutational multivariate analysis of variance (PERMANOVA) were used to evaluate the difference of various niche community structure by R vegan package. Rarefaction was analyzed by Mothur (1.43.0) and visualized using R software (Altschul et al., 1997). Based on the relative abundances of bacteria and fungi at the phylum level, bar charts of the microbial community composition were generated using MS Excel 2016. The flower Venn diagram was constructed with the R “VennDiagram” package (version 1.6.20) (Chen and Boutros, 2 011). The heatmaps of the bacterial and fungal communities at the genus level in the RS and organs of LLPs were generated using OmicShare tools on an online platform^4^ (Yang et al., 2025b; Li et al., 2020), and the correlation heatmaps among the microbial communities and the parameters of the soil, plants, and fruits were also generated with the OmicShare tools (Huang et al., 2023) based on Pearson correlation coefficients, after the abundance data of microbial genera were subjected to normal distribution tests.

### 2.9 Isolation and validation of LLP pathogenic fungi

The validation experiments of pathogenic fungi were carried out following Koch’s rule, including pathogen isolation, culture, introducing into healthy LLP seedling, identification, and tracking of the pathogens in diseased LLP plants. In accordance with the method of Melo et al. (2014), the fungal pathogens were isolated from above diseased LLP leaves, which were stored in -80 °C refrigerator after they were freeze-dried. The diseased LLP leaf samples were cut into about 2.0 cm pieces, watered in running water to remove soil or other particles, then immersed and shook in 75% alcohol (v/v) for 1 min, and then rinsed three times in sterilized deionized water. After the sterilized leaf pieces were dried with sterilized tissue paper, they were put on the potato dextrose agar (PDA) with 80 mg L^–1^ antibiotics (tetracycline) for inhibiting the growth of bacteria, four leaf pieces each Peri dish (Abo-Elyousr et al., 2024). The PDA dishes with leaf pieces were incubated in darkness for 1–2 days at 28 °C. After the hyphal tip of the fungal strain generated from the leaf pieces, they were transferred to the central on a new PDA dish by hypha tip isolation (Brown, 1924) and incubated under the same condition as before, the purified fungi were individually transferred to slants, then stored at 4 °C for validation experiments.

long line pepper seedling cultivation and pathogenic incubation were carried out. Specifically, LLP seeds were same as the seeds of the collected pepper in field investigation. To eliminate a variety of pathogen on the surface, the pepper seeds were soaked in 55 °C sterile warm-water for 20 min, during which continuous stirring was required, then the seeds were taken out and soaked in sterile water for 10 h to ensure that they fully absorb water and expand. After drying the surface water, the seeds were wrapped in a sterile wet cloth, and placed in the condition at 28 °C for germination 3–5 days, during which the seeds should be checked daily and stirred appropriately to ensure were heated evenly to gain higher germination rate, when about 70% of the seeds began to germinate, they could be sown in seedling cultivation tray with nutrient soil in a greenhouse with natural light. When the pepper seedling grew up to about 8 cm, which were transferred to pots (one seedling a pot), each pot (8 cm × 10 cm) contained 1.5 kg mixed humans and calcareous yellow soil (1:1, W/W) (Abo-Elyousr et al., 2024; Cai et al., 2025). After the seedlings grew for 5 days, 2 mL pathogenic fungal spore suspension liquid of an isolate (10^5^ CFU mL^–1^) was inoculated to the root of a seedling for infection experiment, which was put into a 30 cm × 30 cm × 30 cm space for separation culturation, in order to avoid inter-infection of different pathogens or treatments. Five repetitions a strain, Control seedling was treated with 2 mL sterile water (Wang et al., 2023). All pathogenic infection experiments were carried out in a greenhouse with natural light and relative humidity, about 23 °C –28 °C. After cultivation for 14 days, the pathogenicity symptoms consist with the previous LLP in investigation field were recorded and chlorophyll concentrations of diseased seedling leaves were determined. The pathogenic fungi were selected to be identified.

The Sequencing of the isolated fungal pathogens were submitted to Sangon Biotechnology Co., Ltd (Chengdu City, China). The trains were transported in a foam box with dry ice to the Sangon laboratory. The pathogenic DNA was extracted using Ezup Column Fungal Genomic DNA Purification Kit (B518259, Sangon Biotechnology, Chengdu, China), following the manufacture’s instruction. The common primer pair ITS1(5’-TCCGTAGGTGAACCTGCGG-3’) and ITS4 (5’-TCCTCCGCTTATTGATATGC-3’) were applied to amplify ITS region of fungal rDNA, and the PCR product length was 600 bp (Yang et al., 2025a). A 25 μL reaction mixture of amplification system contained template including extracted fungal DNA), primer up (ITS1), primer down (ITS4), dNTP mix, 10 × Taq reaction buffer, Taq polymerase, and deionized water (Supplementary Table 3). The procedure of PCR amplification was the following: pre-denaturation at 95 °C for 5 min, followed by 35 cycles of denaturation at 94 °C for 30 s, annealing at 57 °C for 30 s, extension at 72 °C for 90 s, repair and extension 72 °C for 8 min, at last termination at 4 °C. The purified PCR products were sequenced on the Illumina Miseq platform. The sequences of pathogenic fungal ITS were aligned using Blast of the NCBI website.^5^ The phylogenetic trees of isolates were generated by adjacency method with MEFA11 software, at last the species of fungal isolates were identified. The following special primers of the both pathogens were designed according to the their ITS sequences which were list in Supplementary Table 4.

The fungal relative biomass of pathogensYW25 and YW28 in infected pepper seedling roots were measured with DNA-based quantitative PCR (qPCR) according to the 2^−ΔΔCT^ method (Livak and Schmittgen, 2001). In short, the total DNA was isolated according to the Cetyltetramethyl ammonium bromide (CTAB) method (Murray and Thompson, 1980) and primer pair qYW25/28F + qYW25/28R was used to amplify fungal DNA, whereas the *Tublin*2 gene was used as an internal standard (Qiao et al., 2025; Varela et al., 2025). Representative agarose gel electrophoresis of abstracted specific DNA of both fungal pathogens from pepper seedlings treated by YW25 and YW28 were presented in Supplementary Figure 4. The reaction system of real-time qPCR (RT- qPCR) included ChamQ SYBR Green qPCR master Mix (Vazyme biotechnology Co., Ltd, Nanijing, China), primers, Template (DNA), and ddH_2_O (Supplementary Tables 5, 6). RT-qPCR procedure followed: pre-denaturation at 95 °C for 30 s, followed by 40 cycles of denaturation at 95 °C for 10 s and annealing at 60 °C for 30 s. The purified RT-qPCR products were determined using Fluorescence quantitative PCR Analyzer (Sichuan Jielai Mei Technology Co., Ltd, Chengdu, China).

## 3 Results

### 3.1 Soil physicochemical properties and enzymatic activities

To investigate the physicochemical properties and enzymatic activities in the RS of healthy and diseased LLP plants, the pH, concentrations of OM, AN, AP, AK, and PHA, SUA, and URA were determined. These RS parameter differences between both LLP health sates were analyzed using *T*-test, the results discovered that no significant differences in the concentrations of soil OM, AN, AP, AK, or PHA were detected between healthy and diseased plants, while the activities of the rhizosphere enzymes SUA and URA from healthy plants were significantly (*P* < 0.05) higher than that from diseased pepper plants, soil pH had the reverse trend (Table 1).

**TABLE 1:** Physicochemical properties and enzymatic activities of soil from LLP plants (*n=3*).

Health state	pH	OM (g kg^–1^)	AK (mg kg^–1^)	AP (mg kg^–1^)	AN (mg kg^–1^)	PHA (mg p-Nit g^–1^ 24 h^–1^)	SUA (mg Glucose g^–1^ 24 h^–1^)	URA (mg NH_4_^+^-N g^–1^ 24 h^–1^)
DI	5.37 ± 0.33a	39.12 ± 0.90a	355.84 ± 53.53a	138.70 ± 1.69a	107.25 ± 13.43a	1.77 ± 0.14a	7.85 ± 0.04b	2.19 ± 0.22b
HE	4.88 ± 0.07b	38.80 ± 3.04a	301.67 ± 19.24a	144.47 ± 2.86a	116.34 ± 1.44a	1.84 ± 0.27a	8.42 ± 0.21a	5.12 ± 0.28a
	*P* = 0.011	*P* = 0.098	*P* = 0.1117	*P* = 0.8718	*P* = 0.453	*P* = 0.820	*P* = 0.049	*P* = 0.001

LLP, long line pepper; The soil OM, organic matter; AN, available nitrogen; AP, available phosphorus; AK, available potassium, PHA, phosphatase activity; p-Nit, paranitrophenol; SUA, sucrase activity; URA, urease activity. The values in the table represent the average of three replicates plus standard error. Different lowercase letters indicate significant differences in soil physicochemical properties or enzyme activity between diseased (DI) and healthy (HE) chili peppers (*P* < 0.05), while the same lowercase letters indicate no significant differences (*P* ≥ 0.05) (*T*-test). *P* presents significant value in the table.

### 3.2 Plant growth and physiology in healthy and diseased pepper plants

**FIGURE 1:**
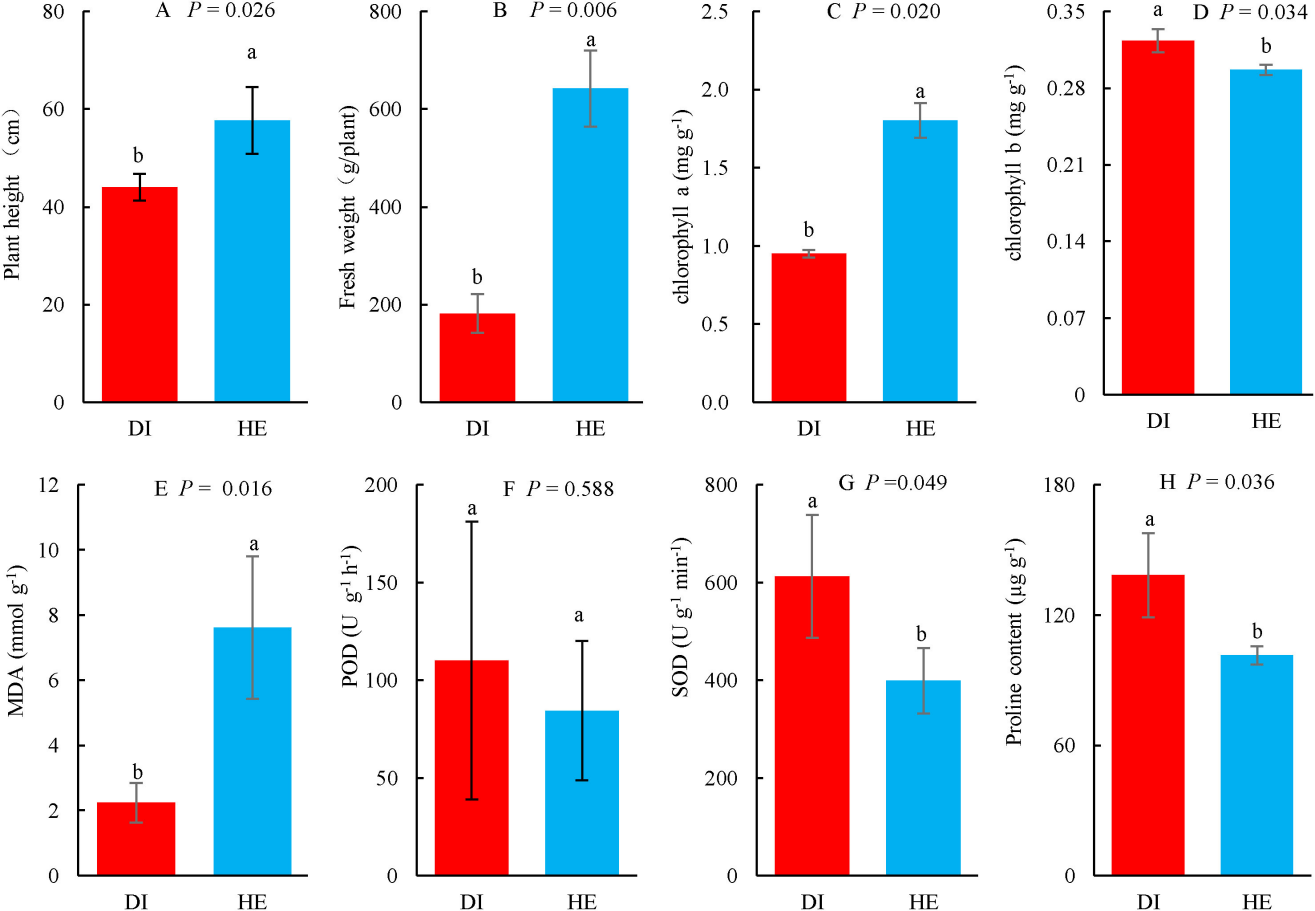
The growth and physiology of long line peppers (LLPs) in diseased and healthy states. **(A,B)** Plant height and fresh weight; **(C–E,H)** Concentration of chlorophyll a, chlorophyll b, MDA malondialdehyde, and proline; **(F,G)** POD peroxidase, SOD superoxide dismutase. Indexes are expressed as average of three repetitions (*n* = 3) plus standard errors. The average values followed by different lowercase letters show significant difference between diseased (DI) and healthy (HE) LLPs at *P* < 0.05, same lowercase letters show no significant difference *P* ≥ 0.05. *P* shows the significant values in figures (*T*-test).

**FIGURE 2:**
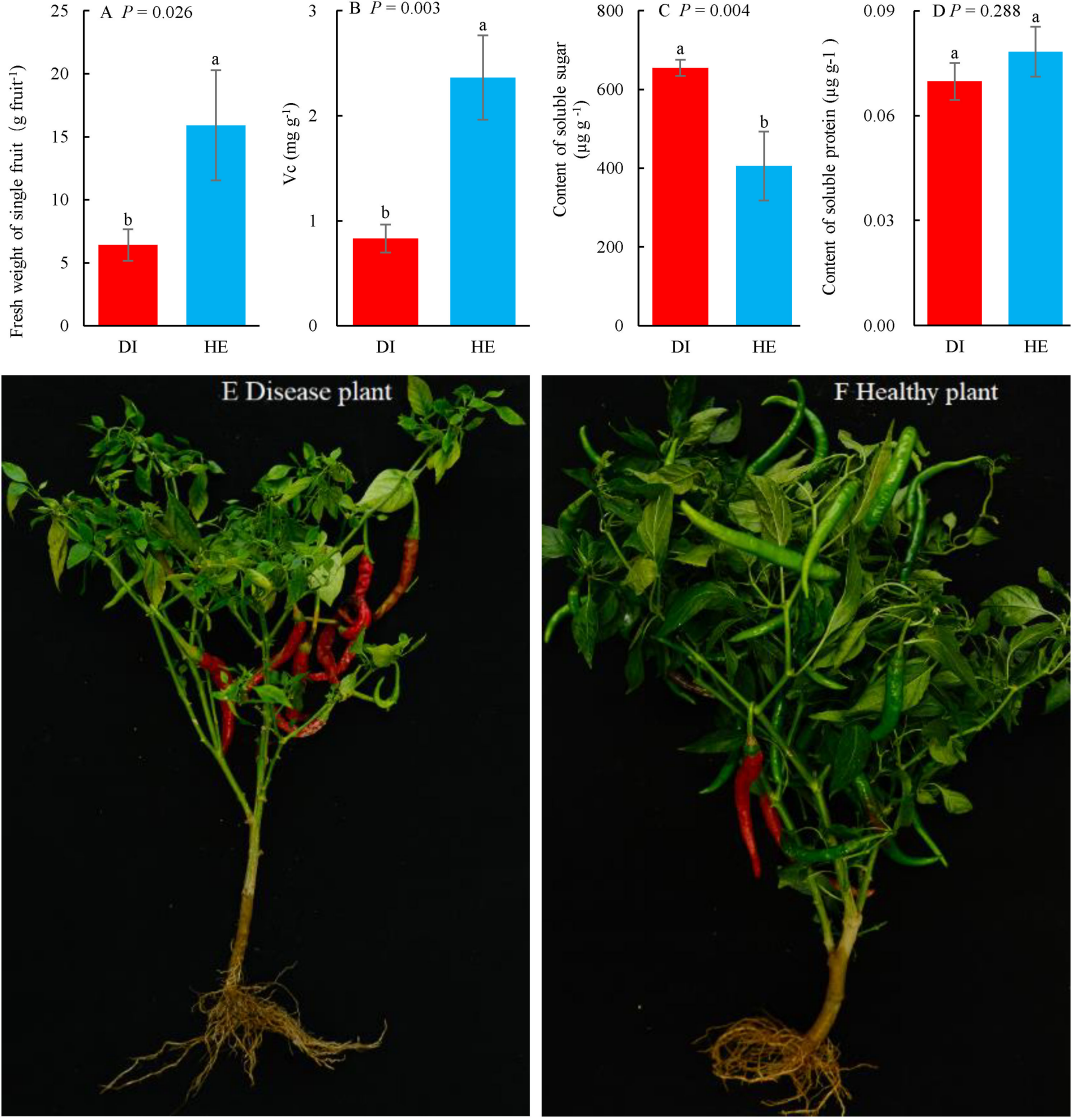
The plant photos, weight of single fruit and quality of long line pepper (LLP) fruits in diseased and healthy states. **(A)** Fresh weight of single fruit; **(B–D)** Concentrations of fruit vitamin c (Vc), soluble sugar and protein, respectively. The parameters are expressed as means of three repetitions plus standard errors (*n* = 3). The average parameters followed by different lowercase letters express significant difference between diseased and healthy LPP fruits at *P* < 0.05, same lowercase letters show no significant difference *P* ≥ 0.05. *P* shows the significant values in figures (*T*-test). **(E,F)** Diseased and healthy LLP.

To evaluate the growth and physiology of the diseased and healthy LLP plants, plant height, fresh weight, concentrations of chlorophyll a, b, proline, and MDA, and activities of the antioxidant enzymes POD and SOD were determined (Figure 1). To investigate the LLP growth and fitness differences in both health states, these variables were subjected to *T*-test, the results showed that plant height, fresh weight, and chlorophyll a and MDA concentrations were significantly lower in diseased plants than in healthy plants (Figures 1A–C, E), while the opposite trend was observed for SOD activity and chlorophyll b and proline concentrations (Figures 1D, G, H). The increasing of SOD activity and proline concentrations individually implied oxidative stress and osmotic stress increased for diseased LLPs under the disease challenges. Although POD activity in the leaves of diseased plants was higher than that in healthy plants, the difference was not significant (Figure 1F). Observations of the phenotypes reported that diseased plants were thinner and had more yellow leaves than healthy plants (Figures 2E, F).

### 3.3 Fruit weight and quality in healthy and diseased pepper plants

The mean fruit weight and quality in healthy and diseased LLP plants were compared (Figure 2). Several fruit parameters, namely the single fresh weight and concentrations of Vc, soluble sugar, and protein differed between plants of the two health states. Based on the *T*-test, the single fruit fresh weight and the Vc concentration were significantly higher (*P* < 0.05) in healthy pepper plants than in diseased pepper plants (Figures 2A, B). In contrast, the soluble sugar concentration in fruits of healthy pepper plants was significantly lower (*P* < 0.05) than that in fruits from diseased pepper plants (Figure 2C), while soluble sugars are key physiological active substances that determine pepper’s disease resistance (Li et al., 2006). The soluble protein concentration in the fruits from healthy plants was higher than that in those from diseased plants, albeit not significantly so (Figure 2D). Moreover, there were more fruits on healthy plants, but a greater proportion of the fruits were red on the diseased plants than on the healthy plants. The fruits in healthy pepper were plumper than those in diseased plants (Figures 2E, F).

### 3.4 DNA sequencing results

**FIGURE 3:**
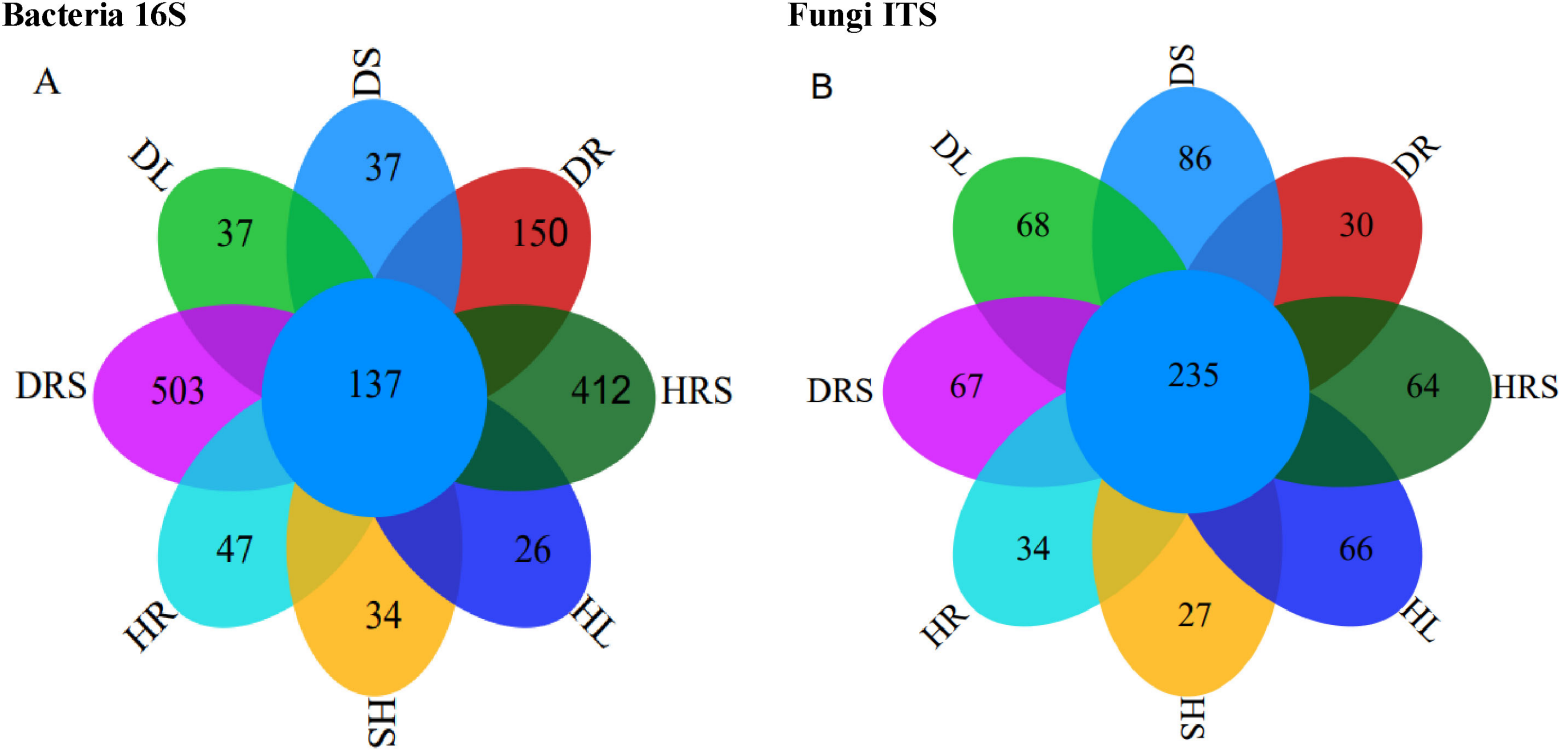
Results of 16S and internal transcribed spacer (ITS) sequencing from the rhizosphere soil (RS), roots, stems and leaves of long line pepper (LLPs), **(A,B)** Operational taxonomic unit (OUT) flower venn diagram about bacterial 16S and fungal ITS, respectively. DRS shows RS from diseased LLP; DL, DS and DR represent the leaf, stem, and root of diseased LLP, respectively. HRS shows RS from healthy LLP; HL, HS, and HR individually represent the leaf, stem, and root of healthy LLP. The same as the following figures.

Raw image data files from Illumina Miseq™ were converted into raw reads. Overall, 1,084,892 raw bacterial and 1,549,107 fungal raw reads were obtained from the 24 soil and plant sub-samples as a result of high-throughput sequencing analysis, with the raw sequence lengths ranging from 39 to 510 bp for bacteria and from 42 to 493 bp for fungi (Supplementary Tables 7, 9). After raw reads were subjected to processing including chimeric filter, mitochondrion, and chloroplast read deletion et al., 1,012,802 clean bacterial and 1,544,854 clean fungal reads were obtained, and the length of the sequences ranged from 350 to 468 bp for bacteria and from 100 to 451 bp for fungi. A total of 15,920 bacterial and 9, 907 fungal OTUs were obtained by clustering at a 97% identity level (Supplementary Tables 8, 10). It suggested that the bacterial species were more enriched and community complex than fungi, additionally, the OTU difference between bacteria and fungi was related to the sequencing depth and primer bias. And the library coverages ranged from 99.44%–99.67% for bacteria to 99.73%–99.87% for fungi, respectively (Supplementary Figures 6A, B). The rarefaction curves of the microbial Shannon indices approached a plateau (Supplementary Figures 5A, B), which indicated that the sequencing depth had reached saturation. To compare the correlations among these fungal or bacterial communities in detail, the shared and sample-specific species were analyzed using a flower Venn diagram (Figure 3). There were 137 bacterial OTUs shared by the eight samples (Figure 3A), which was lower than the number of fungal shared OTUs (235) by all samples (Figure 3B). However, the total bacterial OTU number was higher than that for the fungi (Figures 3A, B). Although a large number of OTUs were detected, there were fewer specific OTUs, and even fewer common OTUs in all samples, implying that fewer common microbial communities in different niches exist stably.

### 3.5 Microbial community structure and diversity

**FIGURE 4:**
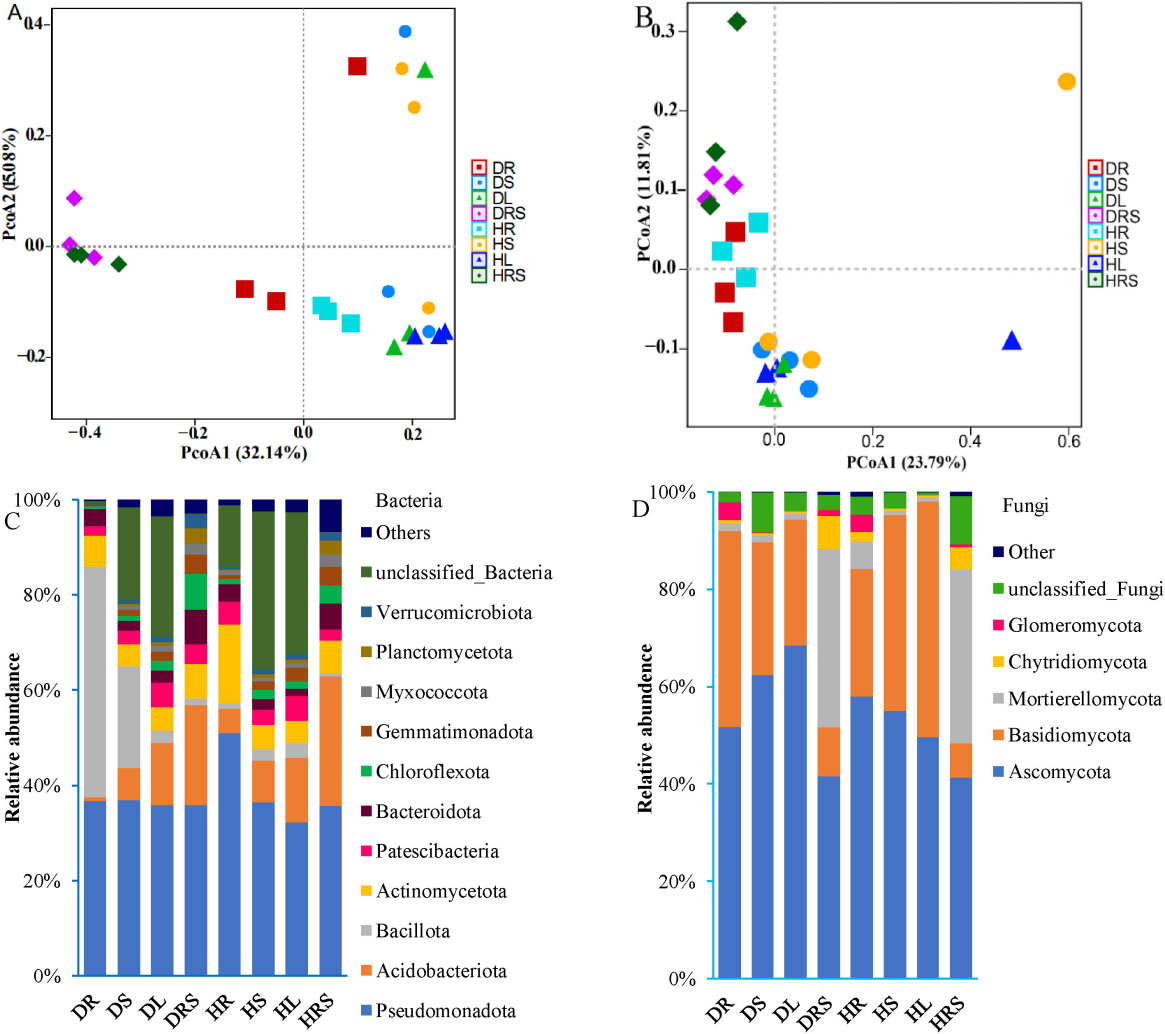
Beta-diversity and phylum composition of bacterial and fungal communities from the rhizosphere soil (RS), roots, stems, and leaves of long line peppers (LLPs). **(A,B)** 16S and internal transcribed spacer (ITS) principal coordinate analysis (PCoA) according to operational taxonomic units (OTUs), respectively, the nearer Bray–Curtis distance is, the more similar the structure is. **(C,D)** Compositions of bacteria and fungi communities at the phylum level, with average relative abundance above 1% of all samples, respectively. Bacterial or fungal community with relative abundance below 1% were assigned as others.

At the phylum level, the dominant bacterial phyla in the RS and organs of LLP plants were Pseudomonadota, Acidobacteria, Bacillota, Actinobacteria, Patescibacteria, Bacteroidota, Chloroflexota, Gemmatimonadota, Myxococcota, Planctomycetota, and Verrucomicrobiota, which accounted for 64.3%–98.7% of the total bacterial abundance in all samples (Figure 4C and Supplementary Table 13). The relative abundance of the bacterial phylum Pseudomonadota ranged from 32.2% to 50.9%, and the highest relative abundance was detected in the healthy root (HR) of peppers, and significantly higher (*P* < 0.05) than those of other samples, followed by diseased stem (DS), root (DR), healthy stem (HS), diseased leaf (DL), diseased rhizosphere soil (DRS), and healthy rhizosphere soil (HRS), while the lowest abundance was in the healthy leaf (HL), no significant difference (*P* ≥ 0.05) was detected among latter seven samples. Bacterial phylum Actinobacteria was the most enriched in RS of both health states, with the relative abundances at 21.1% (DRS) and 27.2% (HRS), there were no significant difference (*P* ≥ 0.05) between both health RS samples, followed by leaves and stems in both health states, the lowest was in DR (0.76%), significant dissimilarities (*P* < 0.05) in its abundance were observed among RS, stem and leaves in healthy or diseased state peppers except for unsignificant difference (*P* ≥ 0.05) between HR and HS samples. Similar to Actinobacteria, the relative abundances of the bacterial phyla, Bacteroidota Chloroflexi, Gemmatimonadota, Myxococcota, Planctomycetota, and Verrucomicrobia were also highest in the RS and differed among the different phyla. However, the bacterial phylum Actinomycetota had the highest abundance in the HR (16.8%), followed by DRS and HRS, and significantly higher (*P* < 0.05) than those of HR, DRS and other samples. Bacillota was the most abundant in the DR (48.4%), followed by DS (21.4%), and was significantly higher (*P* < 0.05) than in the remaining samples DL, DRS, HR, HS, HL, and HRS. Patescibacteria had the highest relative abundance in leaves of both health pepper states (DL 5.2%, HL 5.2%), followed by DRS and HR, no significant differences (*P* ≥ 0.05) were found among these four samples, which were significantly higher (*P* < 0.05) than other four samples (DR, DS, HS, and HRS). It suggested that the relative abundance at bacterial phylum level varied with different phyla and niches.

The relative abundances of fungal phyla varied with differences in the health status and organs of the LLP plants (Figure 4D). The statistical multiple-comparisons among all samples were presented in Supplementary Table 15. The total abundances of the top five fungal phyla, namely the Ascomycota, Basidiomycota, Mortierellomycota, Chytridiomycota, and Glomeromycota, were greater than 89% in all samples, but relative abundance of each in DRS and HRS samples were not significantly different (*P* ≥ 0.05). However, the relative abundance of Ascomycota gradually increased from root (51.7%) to stem (62.4%) and leaf (68.5%) in diseased plants but decreased in the corresponding samples of healthy plants (57.9% for the HR, 55.0% for the HS, and 49.5% for the HL). The abundances of this phylum in DS and DL samples were significantly higher (*P* < 0.05) than those of DR, DRS, HL, and HRS samples, no significant differences (*P* ≥ 0.05) between the both DS and DL, among the four DR, DRS, HL, and HRS samples were discovered. Unlike the abundance of Ascomycota, the relative abundance of Basidiomycota gradually decreased from the DR (40.2%) and DS (27.2%) to DL (25.8%); conversely, it gradually increased in healthy pepper roots (26.3%), stems (40.1%) and leaves (48.4%). Its abundances were not significantly different (*P* ≥ 0.05) among DR, HS, and HL samples, but significantly higher (*P* < 0.05) than in other samples, the lowest in plant RS and no significant difference (*P* ≥ 0.05) between both RS samples was found. The opposing trend in Basidiomycota and Ascomycota abundances might be due to their different adaptability for various niches and bioactive compound synthesis. Mortierellomycota exhibited the highest relative abundances in the RS (DSR, 36.7%; HRS, 35.8%), followed by roots, stems, and leaves, while the relative abundance of Mortierellomycota in the DR (1.7%) was significantly lower (*P* < 0.05) than that in HR (5.6%). The variation in the abundance of Chytridiomycota was similar to that of Mortierellomycota, but its abundance was lower than that of Mortierellomycota in all samples. The relative abundance of Glomeromycota was highest in the roots, followed by the RS, while its abundance in the stems and leaves did not exceed 0.1%. All the relative abundances of bacterial and fungal communities at phylum level were analyzed using PERMANOVA test, the result showed that the significant dissimilarity (*P* < 0.05) existed among all the samples in diseased and healthy LLP plants (Supplementary Tables 14, 16).

To evaluate the microbial community structure in diseased and healthy LLP plants, bacterial and fungal PCoA were performed based on Bray–Curtis distance coefficients (Figures 4A, B). The results revealed that the contribution rates of PCoA1 were higher than those of PCoA2, and the contribution rates of cumulative variances were 47.22% for bacteria and 35.60% for fungi; the closer the distance between groups, the more similar the communities are. Bacterial PCoA1 visually separated the RS from the roots, stems, and leaves in the community structure, while fungal PCoA2 generally distinguished the RS communities from those in roots, stems, and leaves, and the bacterial and fungal communities of the roots were distinguishable from those of the stems and leaves. And the bias was furtherly determined using PERMANOVA test, the difference in bacterial and fungal community structure among all samples was significant (*P* < 0.05) (Supplementary Tables 11, 12).

**FIGURE 5:**
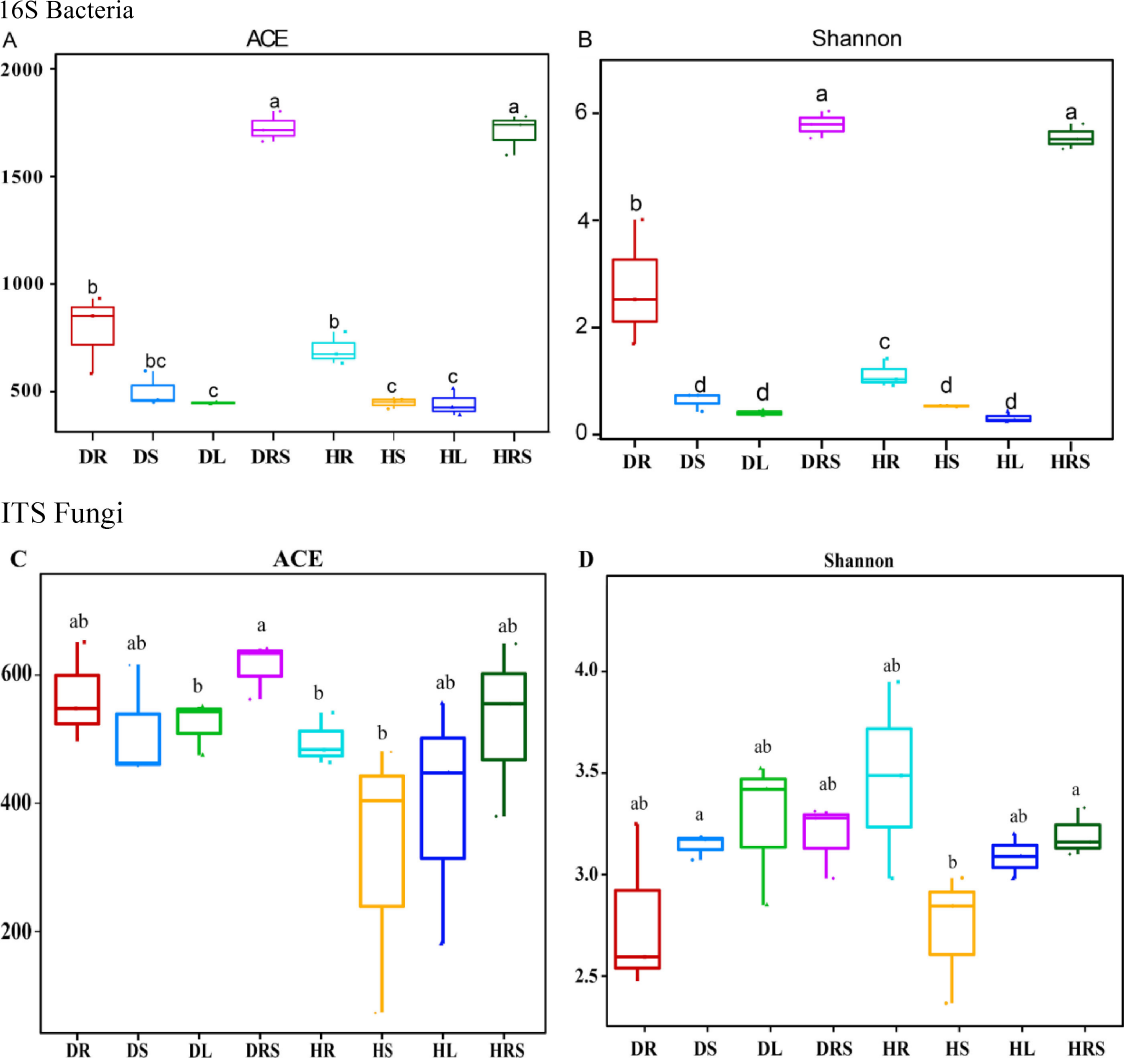
Alpha diversity of bacterial **(A,B)** and fungal **(C,D)** communities from the rhizosphere soil (RS), roots, stems, and leaves of long line peppers (LLPs). Different lowercase letters show significant difference between two samples at *P* < 0.05, identical lowercase letters show no significant difference between two samples (*P* ≥ 0.05) [One-way analysis of variance (ANOVA) and Least Significant Difference (LSD) test].

The microbial α-diversity ACE index was used to evaluate the microbial richness of all samples, and the Shannon index was adopted to estimate the microbial species diversity of all samples in the two health states. As for bacteria, the results showed that the ACE index of the RS was the highest, but no significant difference was detected (*P* ≥ 0.05) between the DRS and the HRS. Moreover, the ACE indices of the RS from diseased and healthy pepper plants were significantly higher (*P* < 0.01) than those in the other samples, followed by the ACE indices of the roots. The ACE index of the DR was higher than that of the HR, although no significant difference (*P* ≥ 0.05) was found between the two samples. The ACE indices of the stems and leaves (except for that of the DS) were significantly lower (*P* < 0.05) than those of the roots but there were no differences among the DS, DL, HS, and HL samples (Figure 5A). The changes in the bacterial Shannon indices in response to disease were similar to those in bacterial ACE, except for the DS, which was significantly different (*P* < 0.05) from the HR (Figure 5B). However, the fungal α-diversity changes in ACE and Shannon indices were different from those of bacteria, but no significant differences (*P* ≥ 0.05) were found between any samples, except for the ACE index in the DRS, which was significantly higher (*P* < 0.05) than those in the DL, HR, and HS samples, and Shannon index in HS which was significantly lower (*P* < 0.05) than DS and HRS samples (Figures 5C, D).

### 3.6 Bacterial and fungal community differences at the genus level

**FIGURE 6:**
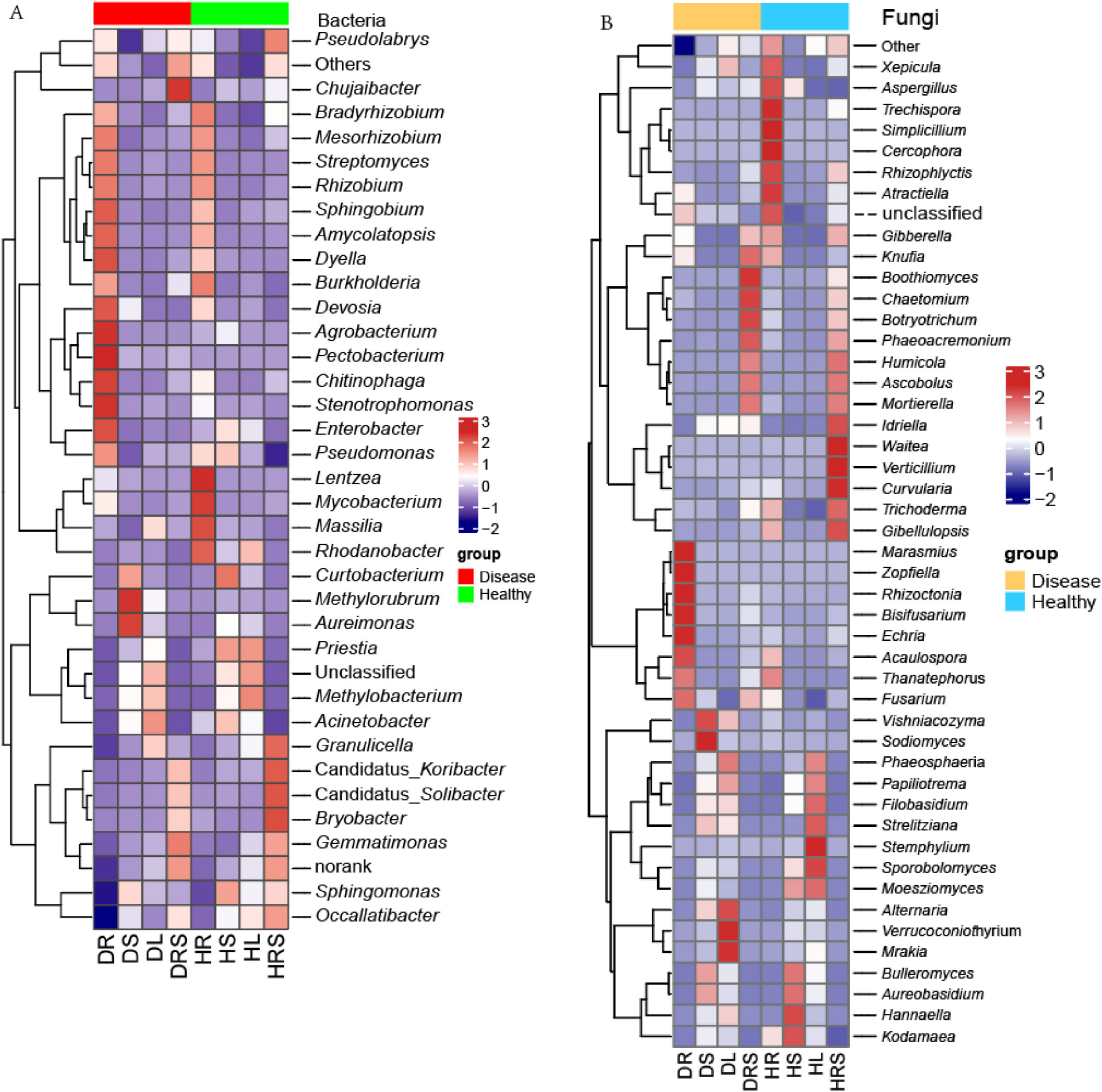
The heatmaps of the bacterial and fungal communities at the genus level with the average relative abundance above 0.1% of all samples. **(A)** Heatmap of bacteria; **(B)** Heatmap of fungi. Normalization and classification were conducted in row.

To obtain a broad perspective of how the microbial communities in the RS and vegetative organs of LLP plants varied in response to disease stress, a relative abundance heatmap of the microbial communities among the eight niches was generated using row cluster analysis (Figure 6). Bacterial and fungal heatmaps showed the top 34 and 46 communities, respectively, with more than 0.1% average relative abundance of all samples. Simultaneously, to qualify the abundances of microbes, dot-rod heatmaps were generated (Supplementary Figures 7, 8). As for bacteria, the top four dominant genera with average relative abundance above 2% were *Sphingomonas*, *Acinetobacter*, *Methylobacterium*, and *Rhodanobacter* (Supplementary Figure 7). The bacterial genus *Sphingomonas* predominated in the vegetative stem, with mean relative abundance 11.3%, its abundance was the highest in HS, followed by DS, HRS, HL, and DL, the lowest was in DR, moreover, its abundance was not significantly different (*P* ≥ 0.05) among HS, DS, and HRS samples and between HR and DR samples, but the abundances in the former three sample were significantly higher (*P* < 0.05) than those of the latter two samples. The second-most enriched bacterial genus, *Acinetobacter* had a mean relative abundance 6.4%, ranging from 0.09% (HRS) to 14.7% (DL), its abundance trend was DL > HS > DS > HL > HR > DRS > DR > HRS, and no significant differences (*P* ≥ 0.05) among DS, HL and HR samples, and among DR, DRS and HRS were detected. While third-most enriched bacterial genus *Methylobacterium* had a mean relative abundance 2.0%, ranging from 0.03% (HRS) to 7.7% (HL), the abundance level trend was HL > DL > HS > DS > HR > DR > DRS > HRS, and its abundance in HL was significantly higher (*P* < 0.05) than those in other samples, while there were no significant differences (*P* ≥ 0.05) among DS, DL and HS, and among the latter four samples. *Methylorubrum* had the highest abundance in DS, which was significantly higher (*P* < 0.05) than other samples, followed DL, no significant difference (*P* ≥ 0.05) among other samples except for DL was found. Similarly, *Aureimonas* exhibited same trend. Among all the samples, the abundances of the 13 bacterial genera *Mesorhizobium*, *Chitinophaga*, *Streptomyces*, *Devosia*, *Enterobacter*, *Amycolatopsis*, *Stenotrophomonas*, *Pectobacterium, Dyella*, *Sphingobium*, *Agrobacterium*, *Rhizobium*, and *Pseudomonas* were highest in the DR, followed by HR, and these 13 genera had low relative abundances in the stems and leaves, with the exception of *Agrobacterium*, *Pectobacterium, Enterobacter*, and *Pseudomonas*. The relative abundances of the bacterial genera *Gemmatimonas* and *Chujaibacter* were highest in the DRS, followed by the HRS, the abundances of which were lower in stems and leaves, the lowest was in roots of both health state LLPs. However, *Bradyrhizobium*, *Lentzea*, *Mycobacterium*, *Massilia*, *Rhodanobacter* exhibited the highest relative abundances in the HR across all samples, the former three genera followed by the relative abundance in the DR. Moreover, the abundance of these three genera in the DR sample was significantly higher (*P* < 0.05) than that in the stems and leaves of both health states, and there were no significant differences (*P* ≥ 0.05) among the stem or leaf samples. *Bryobacter*, *Granulicella*, Candidatus_*Koribacter*, and Candidatus_ *Solibacter* shows the highest abundance in HRS sample, the lowest in DR, and had no significant differences (*P* ≥ 0.05) in the four genus abundances among roots, stems and leaves except for the HL (Supplementary Figure 7 and Figure 6A). These findings suggested that the relative abundances and composition of bacterial communities were varied with dissimilar genera and niches. In addition, there were specific microbial taxa uniquely associated with a particular plant niche, just like the bacterial genus *Angustibacter* was only detected in the RS with the relative abundance of 0.03%.

Among the fungal genera, *Aureobasidium* predominated in LLP plants based on the average relative abundance (10.8%) across all samples, and the relative abundance in the stems was the highest, followed by leaves, and its relative abundance in the stems and leaves was significantly higher (*P* < 0.05) than that in the RS and roots. The highest average abundance of *Aureobasidium* was followed by those of the fungal genera *Mortierella* (10.5%)*, Sporobolomyces* (6.6%), *Alternaria* (6.6%), and *Thanatephorus* (4.9%) (Supplementary Figure 8). Among all the samples, the relative abundances of the fungal genera *Fusarium*, *Marasmius, Rhizoctonia*, *Zopfiella*, *Acaulospora*, *Bisifusarium*, *Echria*, and *Thanatephorus* were highest in the DR, varied among the different samples, and their abundances in DR were significantly higher (*P* < 0.05) than those of other samples except for no significant difference (*P* ≥ 0.05) in *Thanatephorus* abundance between DR and HR samples. *Vishniacozyma* and *Sodiomyces* were most abundant in the DS, with the abundance in the DS being significantly higher (*P* < 0.05) than in any other samples. *Alternaria*, *Verrucoconiothyrium*, and *Mrakia* had the highest relative abundance in the DL; the relative abundances of five fungal genera *Chaetomium*, *Boothiomyces*, *Botryotrichum*, *Knufia*, and *Phaeoacremonium* were highest in the DRS, followed by the HRS, except for *Knufia*, which exhibited the second-highest abundance in the HR. *Simplicillium*, *Rhizophlyctis*, *Cercophora*, *Trechispora*, *Xepicula*, *Aspergillus*, and *Atractiella* were most abundant in the HR, the preceding genera were followed by the HRS, and the following three genera were individually followed by the DL, HS, and DR. *Aureobasidium*, *Bulleromyces*, *Hannaella*, and *Kodamaea* genera were most abundant in the HS. The relative abundances of the genera *Sporobolomyces*, *Filobasidium*, *Phaeosphaeria*, *Moesziomyces, Papiliotrema*, *Strelitziana*, and *Stemphylium* were highest in the HL, while the *Waitea*, *Gibellulopsis*, *Curvularia*, *Idriella*, *Verticillium*, and *Trichoderma* genera exhibited the highest relative abundances in the HRS, and significantly higher (*P* < 0.05) than in the other samples. Additionally, the relative abundances of the three genera *Phaeoacremonium*, *Ascobolus*, *Humicola*, and *Mortierella* in the DRS and HRS were significantly higher (*P* < 0.05) than those in the other samples, with no significant differences (*P* ≥ 0.05) between the two health states. *Gibberella* had higher abundant in the HR and HRS and DRS, and no significant difference (*P* ≥ 0.05) among these three samples, their relative abundances were significantly higher (*P* < 0.05) than those of other five samples. It was clear that the relative abundance of the different microbial communities varied among the various organs and health states (Figure 6B and Supplementary Figure 8).

### 3.7 Relationships between microbes and pepper agronomic performance

**FIGURE 7:**
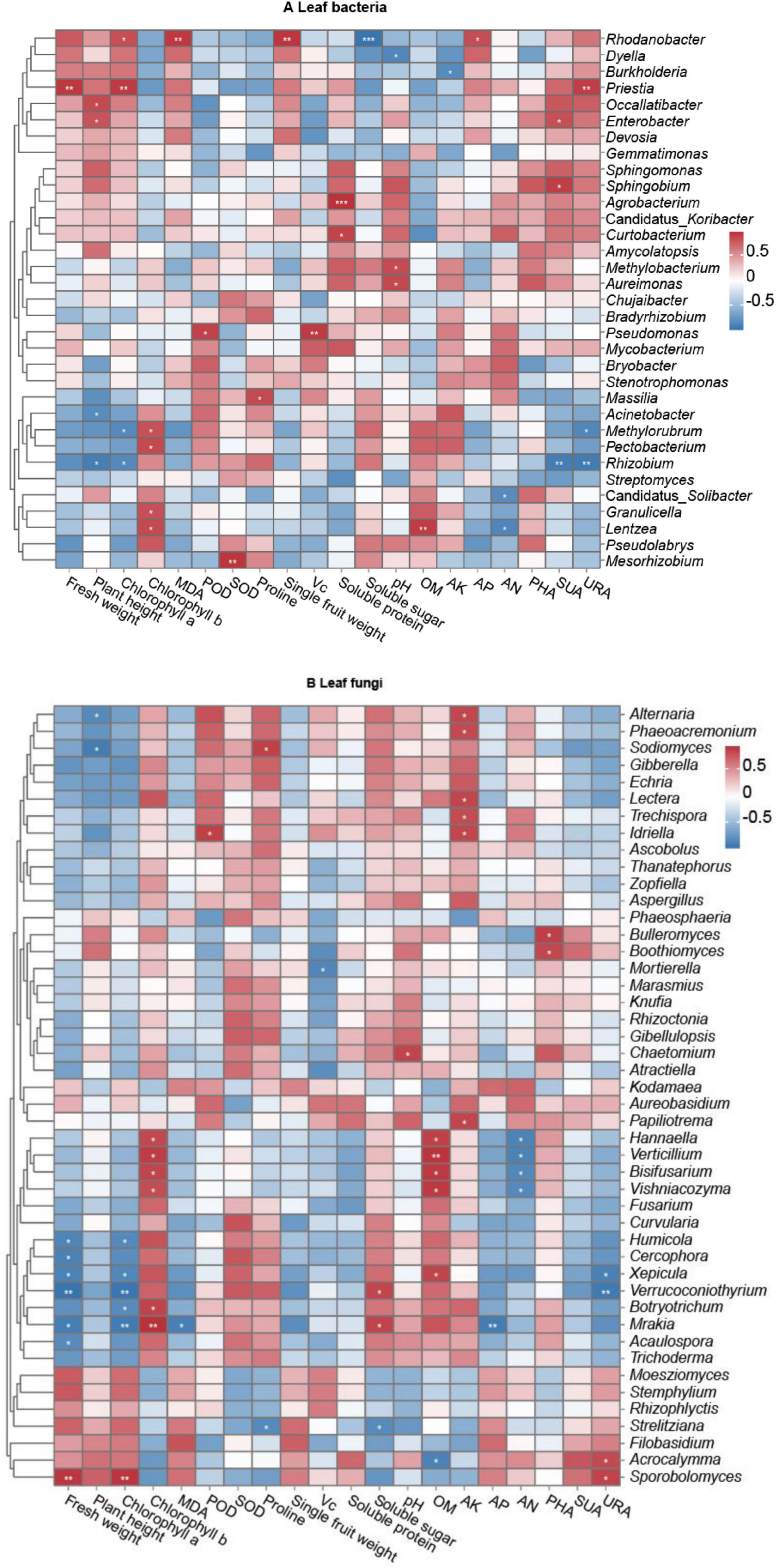
Correlations of long line pepper (LLP) parameters and relative abundances of leaf microbial communities at the genus level. **(A,B)** Leaf bacteria and fungi. No significance *P* ≥ 0.05 was not labeled with *; significance *P* < 0.001 was labeled with ***; **P < 0.01, *P < 0.05. Pearson coefficient values (*r*) were available in Supplementary Figures 8, 9. Normalization and classification were conducted in row.

Using Pearson’s correlation analysis, relationships were determined between microbial genera and pepper agronomic performance parameters (including plant height, fresh weight, leaf chlorophyll, proline and MDA concentrations; plant POD and SOD enzyme activities; mean fresh fruit weight and fruit quality parameters), and soil property parameters, including soil enzyme activities PHA, SUA, and URA (Figure 7 and Supplementary Figures 9–11). The results showed that the abundance of leaf bacterial genera *Rhodanobacter* was significantly positively correlated with leaf chlorophyll a (*P* < 0.05, *r* = 0.838), MDA (*P* < 0.01, *r* = 0.943) concentrations. Bacterial genus *Priestia* was significantly positively related to plant fresh weight (*P* < 0.01, *r* = 0.949), leaf chlorophyll a concentration (*P* < 0.01, *r* = 0.935). The plant height was significantly positively linked with the relative abundances of *Occallatibacter* (*P* < 0.05, *r* = 0.860) and *Enterobacte*r (*P* < 0.05, *r* = 0.815), were significantly negatively linked with *Acinetobacter* (*P* < 0.05, *r* = −0.825) and *Rhizobium* (*P* < 0.05, *r* = −0.911). The concentration of leaf chlorophyll b exhibited significantly positive correlations with the relative abundances of *Methylorubrum* (*P* < 0.05, *r* = 0.830), *Pectobacterium* (*P* < 0.05, *r* = 0.866), *Granulicella* (*P* < 0.05, *r* = 0.833), and *Lentzea* (*P* < 0.05, *r* = 0.864). Additionally, significantly positive relationships were detected between *Pseudomonas* abundance and leaf POD activity (*P* < 0.05, *r* = 0.901), and between *Mesorhizobium* and leaf SOD activity (*P* < 0.01, *r* = 0.960), and between *Mesorhizobium* abundance and leaf SOD (*P* < 0.01, *r* = 0.960). Therefore, pepper growth and health are clearly impacted by certain bacteria. Some bacteria had a positive effect on pepper fruit quality: for example, the concentration of fruit soluble protein was significantly correlated with the relative abundance of *Agrobacterium* (*P* < 0.001, *r* = 0.976) and *Curtobacterium* (*P* < 0.05, *r* = 0.896). In addition, significantly positive correlations were found between single fruit weight and *Rhodanobacter* abundance (*P* < 0.01, *r* = 0.956), and between fruit Vc concentration and *Pseudomonas* (*P* < 0.01, *r* = 0.935). Reversely, the abundance level of *Rhodanobacter* was significantly negatively related to the fruit soluble sugar (*P* < 0.001, *r* = 0.978). The relative abundances of some bacteria were closely related to soil characteristics, with bacterial genus *Dyella* abundance being significantly negatively correlated (*P* < 0.05, *r* = –0.861) to pH value, whereas the relative abundances of *Aureimonas* and *Methlobacterium* were significantly positively correlated (*P* < 0.05, *r* = 0.869; *P* < 0.05, *r* = 0.859) with pH value, and soil AN concentration was significantly negatively correlated with the abundances of Candidatus_*Solibacter* (*P* < 0.05, *r* = −0.821) and *Lentzea* (*P* < 0.05, *r* = −0.861). The rhizosphere SUA was significantly positively related to the abundances of *Enterobacte*r (*P* < 0.05, *r* = 0.834) and *Sphingobium* (*P* < 0.05, *r* = 0.915), whereas the index was significantly negatively related to *Rhizobium* (*P* < 0.01, *r* = −0.918). The rhizosphere URA exhibited significantly positive relationship with *Priestia* abundance (*P* < 0.01, *r* = 0.918), whereas the parameter exhibited significantly negative relationship with the abundances of *Methylorubrum* (*P* < 0.05, *r* = −0.855) and *Rhizobium* (*P* < 0.01, *r* = −0.937). Additionally, significant positive correlations were observed between *Lentzea* relative abundance and soil OM concentration (*P* < 0.01, *r* = 0.925) and between *Rhodanobacter* abundance and soil AP concentration (*P* < 0.05, *r* = 0.842) (Figure 7A and Supplementary Figure 10).

For leaf fungi, the relative abundances of several fungal genera were closely correlated with pepper growth and fruit quality (Figure 7B). The abundances of fungal genera *Humicola*, *Cercophora*, *Xepicula*, *Verrucoconiothyrium*, *Botryotrichum*, *Mrakia*, and *Acaulospora* were significantly negatively correlated with plant fresh weight (*P* < 0.05 or *P* < 0.01, *r* = −0.867, −0.885, −0.876, −0.973, −0.910, and −0.820, respectively) and chlorophyll a concentration (*P* < 0.05 or *P* < 0.01, *r* = −0.831, −0.847, −0.965, −0.818, and −0.935, respectively), with the exception of non-significant (*P* ≥ 0.05) correlations between *Cercophora* (*r* = 0.808) and *Acaulospora* (*r* = 0.747) abundance and chlorophyll a concentration and between *Botryotrichum* abundance (*r* = 0.791) and plant fresh weight. Relative abundances of *Alternaria* and *Sodiomyces* were significantly negatively correlated (*P* < 0.05, *r* = −0.835 and −0.914) with plant height, and *Mortierella* abundance was significantly negatively correlated (*P* < 0.05, *r* = −0.857) with fruit Vc concentration. In contrast, the relative abundance of the fungal genus *Sporobolomyces* was significantly positively (*P* < 0.05) correlated with plant weight and chlorophyll a concentration (*P* < 0.01, *r* = 0.923 and 0.932), while the relative abundances of *Hannaella*, V*erticillium*, *Bisifusarium*, *Vishniacozyma*, *Botryotrichum*, and *Mrakia* were significantly positively (*P* < 0.05 or *P* < 0.01, *r* = 0.842, 0.894, 0.863, 0.813, 0.865, and 0.945, respectively) correlated with chlorophyll b concentration. Additionally, significant positive correlations were observed between *Idriella* relative abundance and POD activity (*P* < 0.05, *r* = 0.876), between *Sodiomyces* abundance and proline concentration (*P* < 0.05, *r* = 0.876), and between the relative abundances of *Verrucoconiothyrium* and *Mrakia* and the concentrations of fruit soluble sugar (*P* < 0.05, *r* = 0.850 and 0.857, respectively). There were also some fungal genera closely related to soil characteristics. For example, the abundances of the fungal genera *Hannaella*, *Verticillium*, *Bisifusarium*, *Vishniacozyma*, and *Xepicula* were significantly positively correlated with soil OM concentration (*P* < 0.05 or *P* < 0.01, *r* = 0.877, 0.935, 0.912, 0.908, and 0.859, respectively). The relative abundances of *Alternaria*, *Phaeoacremonium*, *Lectera*, *Trechispora*, *Idriella*, and *Papiliotrema* were significantly positively correlated with soil AK concentration (*P* < 0.05, *r* = 0.849, 0.836, 0.850, 0.826, 0.845, and 0.852, respectively). Significant positive correlations were found between *Chaetomium* abundance and soil pH (*P* < 0.05, *r* = 0.859), between *Bulleromyces* and *Boothiomyces* abundances and PHA (*P* < 0.05, *r* = 0.883 and 0.847), and between *Acrocalymma* and *Sporobolomyces* abundances and URA (*P* < 0.05, *r* = 0.832 and 0.866), whereas the relative abundances of *Hannaella*, *Verticillium*, *Bisifusarium*, and *Vishniacozyma* were significantly negatively correlated with soil AN concentration (*P* < 0.05, *r* = −0.873, −0.875, −0.851, and −0.834, respectively), while significant negative correlations were detected between *Xepicula* and *Verrucoconiothyrium* relative abundances and the soil enzyme URA (*P* < 0.05, *r* = −0.889; *P* < 0.01, *r* = −0.939), and between *Mrakia* relative abundance and AP concentration (*P* < 0.01, *r* = −0.919) (Figure 7B and Supplementary Figure 11).

Correlations between microbial α-diversity and plant growth, plant physiological parameters, fruit quality, and soil characteristics were also analyzed (Supplementary Figure 9). Although there were positive or negative relationships among them, the only significant correlations were a positive relationship between the leaf bacterial Shannon index and soil AK concentration (*P* < 0.05, *r* = 0.843), and a negative relationship between the leaf fungal Shannon index and soil AN concentration (*P* < 0.01, *r* = −0.924).

In view of the negative effects of some microbes on LLP agronomic performances, regression analysis between the relative abundance of theses microbial genera and LLP agronomic indexes were generated (Supplementary Tables 17, 18). For bacteria, the regression relationships between *Acinetobacter* abundance and plant height (*y* = −1.6939*x* + 69.552, *R*^2^ = 0.681, *P* < 0.001), chlorophyll a (*y* = −0.067*x* + 2.146, *R*^2^ = 0.454, *P* < 0.001), and the concentration of soluble protein (*y* = −0.000498*x* + 0.080, *R*^2^ = 0.050, *P* < 0.05) were significantly negative. *Mesorhizobium* displayed significant negative regression relationship with plant fresh weight (*y* = −1287.456*x* + 533.01, *R*^2^ = 0.423, *P* < 0.01), chlorophyll a (*y* = −1.828*x* + 1.549, *R*^2^ = 0.226, *P* < 0.01), and soluble protein (*y* = −0.007*x* + 0.075, *R*^2^ = 0.08, *P* < 0.001). Significant negative regression relationships were also revealed between *Pectobacterium* and LLP fresh weight (*y* = −1605.616*x* + 511.284, *R*^2^ = 0.331, *P* < 0.05), plant heigh (*y* = -60.667*x* + 54.590, *R*^2^ = 0.374, *P* < 0.001), chlorophyll a (*y* = -3.471*x* + 1.591, *R*^2^ = 0.482, *P* < 0.01), and soluble protein (*y* = −0.046*x* + 0.077, *R*^2^ = 0.171, *P* < 0.001), Similarly, *Pseudolabrys* and *Rhizobium* had significant negative regression trend with some of agronomic performances (Supplementary Table 17). As for fungi, *Alternaria* abundance exhibited significant negative regression relationships with LLP fresh weight (*y* = −11.964*x* + 603.837, *R*^2^ = 0.354, *P* < 0.05), plant height (*y* = −597*x* + 60.419, *R*^2^ = 0.698, *P* < 0.001), and Chlorophyll a (*y* = −0.025*x* + 1.771, *R*^2^ = 0.467, *P* < 0.01). The regression relationship between *Botryotrichum* and LLP fresh weight (*y* = −9034.598*x* + 728.257, *R*^2^ = 0.625, *P* < 0.01), plant height (*y* = −201.085*x* + 57.875, *R*^2^ = 0.245, *P* < 0.01), Chlorophyll a (*y* = −16.744*x* + 1.963, *R*^2^ = 0.669, *P* < 0.05), and soluble protein (*y* = −1.35*x* + 0.079, *R*^2^ = 0.088, *P* < 0.001). Similar to *Botryotrichum*, fungi *Cercophora*, *Humicola, Mrakia, Sodiomyces, Verrucoconiothyrium*, and *Xepicula* displayed same trend regression relationships with above four plant indexes (Supplementary Table 18). The results implied that these microbial genera were closely linked with reductions of the LLP agronomic performances, and suggested that there might be potential pathogens impacting the LLP growth and fitness. It was necessary to carry out pathogen variation experiment.

The interactions among microbes, which displayed positive and negative effect on agronomic traits of LLP, was analyzed using Pearson coefficient method after the normal distributions of microbial abundances were tested. The results revealed that 28 significant positive interactions and 12 significant negative interactions were detected among the 20 selected microbes (Supplementary Figure 12). For instance, the positive interactions between *Acinetobacte*r and *Rhizobium* (*P* < 0.05), between *Occallatibacter* and *Enterobacter* (*P* < 0.05), and between *Xepicula* and *Humicola* (*P* < 0.01) were significant. Inversely, the significant negative interactions between *Rhodanobacter* and *Alternaria* (*P* < 0.001), between *Sporobolomyces* and *Mrakia* (*P* < 0.05), and between *Priestia* and *Humicola* (*P* < 0.01) were detected. It implied that potential synergistic effect or antagonism among the microbes might be possible.

### 3.8 Validation results of pathogens

**FIGURE 8:**
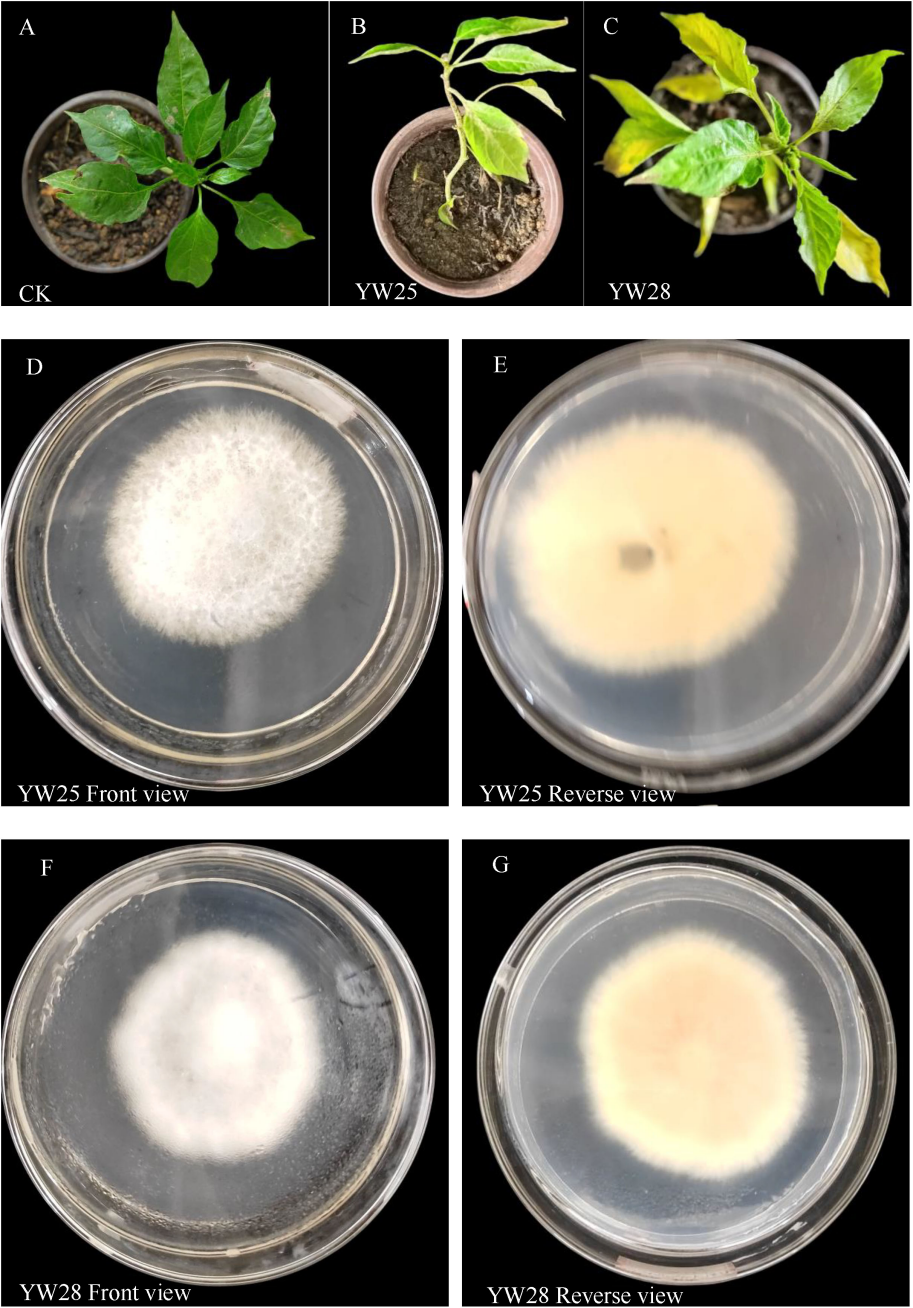
Pepper seedling symptom, community of two pathogenic fungi YW25 and YW28 from the long line pepper (LLP) leaves. **(A–C)** Diseased symptom of pepper seedling after treated by CK, YW25, and YW28 for 14 days. **(D,E)** Community trait of YW25; **(F,G)** Community trait of YW28.

**FIGURE 9:**
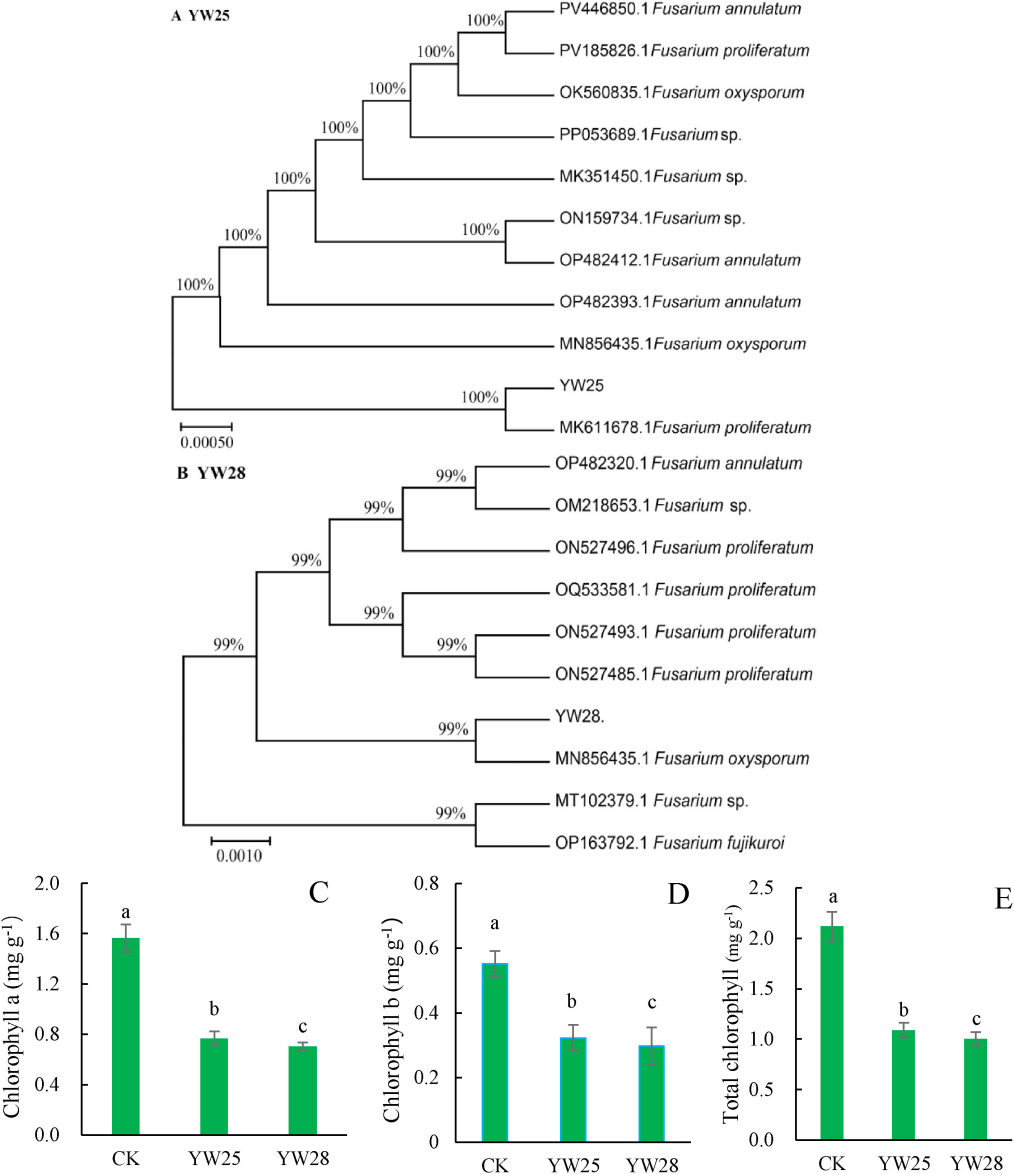
Phylogenetic tree of the two fungal pathogens and chlorophyll concentrations of pepper seedling leaves (*n* = 5). **(A,B)** Phylogenetic tree of the pathogenic isolates. **(C–E)** Concentrations of chlorophyll a, chlorophyll b and total chlorophyll after they were treated by CK (water), YW25 and YW28 for 14 days, different lowercase letters show significant difference between two treatments at *P* < 0.05, identical lowercase letters show no significant difference between two treatments (*P* ≥ 0.05) [One-way analysis of variance (ANOVA) and Least Significant Difference (LSD) test].

Two fungal pathogens were screened from diseased LLP plant leaf, the infected pepper leaves became yellow (Figures 8B, C) and exhibited the disease symptom same as the diseased plant from field investigation (Figure 2E). After ITS sequencing and alignment By Blast against NCBI website, they belonged to two different species of *Fusarium* genus, namely *F. proliferatum* (YW25) and *F. oxysporum* (YW28) (Figures 8D–G, 9A, B). After pepper seedlings were inoculated with YW25 and WW28 for 14 days, respectively, they displayed typical symptoms: the leaves turned yellow from bottom to top. The analysis results of validation experiments showed that these two fungal pathogens caused the significant concentration decreasing of leaf Chlorophyll a, b and total Chlorophyll contrast to CK (*P* < 0.05), but the differences between YW25 and YW28 were not significant (*P* ≥ 0.05) in Chlorophyll a and total concentrations (Figures 9C–E). To further determine the biomass of both pathogenic fungi in infected pepper plants, their relative biomass in pepper root was analyzed using RT-qPCR. The results showed that the relative biomasses of both fungi in seedling roots infected by YW25 and YW28 were 253.14 and 244.57 times of CK treatment, respectively, but no significant difference between both fungi was detected (Table 2). It suggested that these two pathogenic fungi caused pepper yellow-leaf wilt disease and significantly affected the synthesis of Chlorophyll, and their pathogenicity was similar.

**TABLE 2:** The relative biomass of fungal pathogen YW25 and YW28 in pepper seedling roots by real time quantitative polymerase chain reaction (RT-qPCR) analysis.

CK	YW25	YW28
1.00 ± 0.04b	253.14 ± 19.69a	244.57 ± 21.86a

Different lowercase letters show significant difference between two treatments at *P* < 0.05, identical lowercase letters show no significant difference between two treatments (*P* ≥ 0.05) [One-way analysis of variance (ANOVA) and Least Significant Difference (LSD) test].

## 4 Discussion

Crops are subjected to various stresses, such as undesirable conditions of soil (such as nutrient deficiency, inadequate water, and salinization), weather (e.g., waterlogging and drought), and attack by pests and pathogens (Chakraborty and Newton, 2011; Farh et al., 2018). The agronomic traits of crops, such as plant growth and height, physiological properties, and fruit quality, are affected by plant diseases to varying degrees. Moreover, in the same field of a pepper plantation, some pepper plants may suffer from diseases, while others may remain healthy. High-throughput sequencing results from the current study revealed that niche differences occurred in terms of microbial community diversity, structure, and composition among RS, roots, stem, and leaves of diseased and healthy LLP, and the abundance of specific bacterial and fungal communities were closely related to the agronomic performance of the pepper plants.

### 4.1 Potentially pathogenic microbial communities associated with pepper disease

Microbes associated with pepper diseases include pathogenic viruses, fungi, and bacteria. For example, pepper yellows disease, which is caused mainly by the pepper vein yellows virus, results in leaf interveinal yellowing, severe upward leafroll, and even stunted growth (Ahmad et al., 2018; Lotos et al., 2017). Fungal pathogens can lead to disease symptoms such as leaf blight (caused by *Drechslera bicolor*), fruit rot (caused by *Fusarium* spp.), and leaf spots (caused by *Cladosporium subuliforme*), ultimately reducing the yield and shelf life of pepper fruits (Jadon et al., 2016; Ramos-Garcia et al., 2016; Cheon et al., 2015; Utkhede and Mathur, 2003). In current experiment, existent possibility of potential pathogens was defined mainly on the basis of correlation and regression analysis between the relative abundance of microbial communities at the genus level and LLP agronomic performances, if a significantly negative correlation was demonstrated for any microbial genus, then the disease reports on the genus from previous study were referred to as potential pathogens (microbes having negative impact on LLP), which is speculative. Of pepper bacterial diseases, *Pseudomonas* leaf spot disease caused by *Pseudomonas syringae* pv. *syringae* is a seed-borne phytopathogen, and infection by this bacterial pathogen can severely decrease the marketable yield of peppers under suitable conditions and cause significant economic losses (Ranjit et al., 2023). Other *Pseudomonas* species (such as *P. putida*) also cause diseases of pepper fruits and leaves (Kizheva et al., 2022). In the present study, it was determined that the microbial community has a negative impact on the health and fruit quality of LLP plants as reflected in the correlation heatmap and regression relationships between the abundances of microbial communities at the genus level and pepper indicator traits, and possible pepper pathogens were speculated in combination with previous reports of these microorganisms. It was found that the bacterial genus *Pseudomonas* in LLP leaves was significantly positively related to plant POD activity (*P* < 0.05, *r* = 0.901), and was closely negatively (*r* = −0.485) correlated with pepper plant height although the correlation was not significant (*P* ≥ 0.5) (Figure 7A and Supplementary Figure 10). Similarly, the abundance of bacterial *Pectobacterium* exhibited negative relationship with LLP growth and fitness although unsignificant correlations between the genus abundances and plant fresh weight and height were detected (*P* ≥ 0.05, *r* = −0.575 and −0.611) (Figure 7A and Supplementary Figure 10), moreover, certain *Pectobacterium* species such as *P. colocasium*, *P. aroidearum* and and *P. carotovorum* were reported to be bacterial soft rot pathogens of taro and potato (Zhou et al., 2022; Naas et al., 2018). Moreover, regression analysis showed the two bacteria were significantly negatively related to LLP fresh weight (*P* < 0.01, *P* < 0.05), plant height (*P* < 0.01, *P* < 0.001), chlorophyll a (*P* < 0.01), and soluble protein (*P* < 0.01, *P* < 0.001), respectively (Supplementary Table 17). Overall, these findings suggested that *Pseudomonas* and *Pectobacterium* impacted the growth and fitness of LLP plants and *Pseudomonas* was more abundant in roots of diseased line peppers than in those of healthy pepper plants (Figure 6A).

Correlation heatmap revealed that the abundance of the fungal genus *Fusarium* was negatively correlated with the fresh weight of pepper plants (*r* = −0.618) and chlorophyll b concentration (*r* = −0.587), although the correlations were not significant (*P* ≥ 0.5) (Figure 7B and Supplementary Figure 11); the highest abundance was in the DR (5.5%), followed by the DRS (4.1%), and these abundances were significantly higher than those in the RS and vegetative organs of healthy plants (Supplementary Figure 8); regression analysis uncovered that the genus from LLP roots was significantly negatively linked with agronomic indexes including fresh weight (*R*^2^ = 0.164, *P* < 0.05), plant height (*R*^2^ = 0.590, *P* < 0.01), chlorophyll a (*R*^2^ = 0.166, *P* < 0.01), and soluble protein (*R*^2^ = 0.849, *P* < 0.001) (Supplementary Table 19). The plant disease might not be very serious, as shown in the photo of the disease symptoms (Figure 2E), but some of genus species (such as *F. culmorum*, *F. graminearum*, *F. poae*, and *F. mycotoxin*) has been reported to be one of the most pathogenic, phytotoxic and toxin-producing of microorganisms worldwide, and plants invaded by members of this fungal genus are characterized by decreased commercial value and consumer health because of the contamination of crops with mycotoxins (Podgórska-Kryszczuk et al., 2022; Ji et al., 2019). The abundance of the leaf fungus genus *Alternaria* was negatively correlated with plant fresh weight, height, and chlorophyll a concentration, and a significantly negative correlation between *Alternaria* abundance and plant height was observed (*P* < 0.05, *r* = −0.835). *Alternaria* abundance was positively correlated with proline concentration (reflecting osmotic stress) and activity of the antioxidant enzyme SOD in spite of no significant differences among them (*P* ≥ 0.05, *r* = 0.712 and 0.800) (Figure 7B and Supplementary Figure 11). Obviously, the fungal genus *Alternaria* impacted the growth and fitness of LLP plants. A previous study reported that fungal *Alternaria iridiaustralis* caused leaf spot or blight disease in *Iris* plants and led to enormous losses in commercial value (Luo et al., 2018). Additionally, many *Alternaria* species are saprotrophic or phytopathogenic that exist ubiquitously in food products and plants and could accumulate toxic metabolites in the edible parts of plants (Cheon et al., 2015; Utkhede and Mathur, 2003; Ranjit et al., 2023). *Xepicula* abundance was significantly negatively correlated (*P* < 0.05, *r* = −0.876 and −0.847) with the fresh weight and chlorophyll a concentration of the LLP plants, indicating that the *Xepicula* community directly affected pepper growth and chlorophyll a synthesis or indirectly affected chlorophyll a degradation). A adation, indirectly impacting the photosynthesis of pepper leaves (Figure 7B and Supplementary Figure 11). A previous study also verified that the fungus *X*. *yifeii* sp. *nov.* led to leaf blight in *Lasia spinosa* (Araceae) plants (Kizheva et al., 2022). The relative abundance of the fungal genus *Verrucoconiothyrium* was also significantly negatively correlated (*P* < 0.01, *r* = −0.973 and −0.965) with pepper fresh weight and chlorophyll a concentration in this study. Species from the genus *Verrucoconiothyrium* were isolated from the plants *Prosopis* sp., *Eucalyptus* sp., *Acacia mangium*, *Rubus idaeus*, and *Nepeta catenaria* (Podgórska-Kryszczuk et al., 2022), but whether this genus causes plant disease has not been reported. Similarly, the relative abundance of *Mrakia* was significantly negatively correlated with fresh weight and chlorophyll a concentration (*P* < 0.05, *r* = −0.910 and −0.935) although the fungal genus was significantly positively (*P* < 0.05) correlated with chlorophyll b and significantly negatively correlated with MDA concentration (Figure 2B and Supplementary Figure 11). In addition, significant negative regression relationships between fungi *Alternaria*, *Xepicula*, *Verrucoconiothyrium*, and *Mrakia* and some agronomic indexes were found (Supplementary Table 18). It suggested that there were various potential pathogens affecting LLP growth and fitness.

### 4.2 Interaction between microbial communities and LLPs under disease stress

The abundance of some genera in the microbial communities showed marked correlations with LLP plants; for example, the abundance of *Rhizobium* was significantly negatively correlated with plant height and chlorophyll a concentration (*P* < 0.05, *r* = −0.911 and −0.842), but, based on previous studies, *Rhizobium*, a Gram-negative soil bacterium hosting functions in nitrogen fixation, is an essential member of the nitrogen cycle (Li and Li, 2023). Similarly, the abundance of bacterial *Methylorubrum* exhibited negative relationship with LLP growth although unsignificant correlations between the genus abundances and plant fresh weight and height were detected (*P* ≥ 0.05, *r* = −0.708 and −0.770) (Figure 7A and Supplementary Figure 10), however, *Methylorubrum* genus was reported to be dominant endophytic bacteria in Rice (Feng et al., 2023). Bacterial genus *Acinetobacter* are widely distributed in humans and environment reservoirs, such as soil, livestock, water, food, and arthropods (Almasaudi, 2018; He et al., 2025). Currently, the majority of the genus *Acinetobacter* species isolated were well-known human pathogens (Qin et al., 2021). In our experiment, this genus exhibited significantly negative effect on the growth and fitness of LLP plant, because its abundance level was significantly negatively related to plant height (*P* < 0.05, *r* = −0.825), and positively related leaf POD activity and proline concentration in spite of no significant relationship among them (*P* ≥ 0.05, *r* = 0.699 and 0.566) (Figure 7A and Supplementary Figure 10). Regression analysis revealed that *Acinetobacter* exhibited significant negative relationships with LLP plant height (*P* < 0.001), chlorophyll b (*P* < 0.001), and soluble protein (*P* < 0.01) (Supplementary Table 17). The finding was inconsistent with the previous study of Xiong et al. (2025), who reported that *Acinetobacter radioresistens* improved the growth *Cucumis sativus* L. contaminated with polystyrene microplastics. It might be due to different *Acinetobacter* and plant species. Whether this bacterium can cause pepper disease needs to be studied in the future. The abundance of fungal genus *Botryotrichum* was also significantly negatively (*P* < 0.05, *r* = −0.818) correlated with the chlorophyll a concentration (Figure 7B and Supplementary Figure 11), meanwhile, significant negative regression relationship between this fungus and five agronomic indexes were detected. Some species of the genus *Botryotrichum* were isolated, but their pathogenicity to plants has not been reported (Ryu et al., 2023).

In contrast, some microbial communities might be potential beneficial for pepper plants based on the correlation analysis and previous references. In this study, the relative abundance of the bacterial genus *Sphingomonas*, which was the most dominant bacterial genus (Supplementary Figure 7), was positively correlated with the plant height and soluble protein concentration of pepper fruit in spite of no significant correlation (*P* ≥ 0.05, *r* = 0.757 and 0.762), showing that this leaf-endophytic genus could improve pepper growth and fruit quality (Figure 7A and Supplementary Figure 10). Similarly, previous studies had shown that several species of *Sphingomonas* could improve the growth of soybean and tomato plants under drought and salinity stress (Halo et al., 2015). Generally, *Rhodanobacte*r, *Dyella, Burkholderia* and *Priestia* had positive effects on pepper growth and fitness quality (Figure 7A and Supplementary Figure 10). Certain species of them were growth-promoting bacteria. For example, *Rhodanobacte*r bacterial genus (e.g., *R. lycopersici* and *R. geophilus*) exhibited plant growth-promoting properties, including siderophore formation, phosphate solubilization, indole-3-acetic acid and ammonia production (Woo et al., 2024), additionally, *R. ginsengiterae* inhibited the growth of fungal pathogens *Fusarium solani* (Huo et al., 2018). Endophytic bacterium *Dyella marensis* produced siderophore (Herrera et al., 2022). *Burkholderia territorii* GP3 isolated from Mount Papandayan soil, and can secrete extracellular lipase and improve conversion of organic carbon (Putra et al., 2019), *Burkholderia cepacian* has the capacity of nitrogen-fixing (Gontijo et al., 2018). Endophytic *Priestia megaterium* could secreted extracellularly resveratrol which was helpful for plant in response to environmental stress and pathogen infection (Zhang et al., 2023). Thereby, these traits of above genera enhanced the growth and immune defense capacity of plants. Additionally, the relative abundances of the bacterial genera *Occallatibacte*r and *Enterobacter* were significantly positively correlated (*P* < 0.05, *r* = 0.860 and 0.815, respectively) with the plant height (Figure 7A and Supplementary Figure 10). Although the relationship of *Occallatibacter* with plants has not been established, *Enterobacter* was almost ubiquitous in nature and could act human pathogen (Rahi et al., 2024), which did not exhibit inhibition of plants, it might be because of different host. The abundances of the leaf fungal genera *Hannaella*, *Verticillium*, *Bisifusarium*, and *Vishniacozyma* were significantly positively correlated with the chlorophyll b concentration of pepper leaves (*P* < 0.05, *r* = 0.842, 0.894, 0.863, 0.813, respectively), which suggested that these fungi could improve the synthesis of chlorophyll b. The abundance of the fungus *Sporobolomyces* also showed a significantly positive (*P* < 0.05) correlation with pepper fresh weight and chlorophyll a concentration (*P* < 0.05, *r* = 0.923 and 0.932), showing that members of the *Sporobolomyces* genus could enhance the plant growth of LLPs. *Sporobolomyces* species are best known for their relationships with plant leaves and grow in many habitats, such as soil, the plant phylloplane, and aquatic systems (Li et al., 2022; Lorenzini et al., 2019; Fatemi et al., 2022). These microbes exhibiting positively or significantly positive relationships with some LLP agronomic traits might be potential beneficial microbes, the validation of their positive effects will be reported in the forthcoming studies.

In addition, the soil properties, including soil enzymatic activities, in pepper RS also had close relationships with the abundance of some microbial communities. For example, the OM concentration was significantly positively related (*P* < 0.01, *r* = 0.925) to the relative abundance of bacterial genus *Lentzea*. the soil AK concentration was positively correlated with the abundances of the bacterial genera *Pseudomonas*, *Aureimonas*, *Acinetobacte*, *Methylorubrum*, and *Pectobacterium* in spite of no significant correlations among them (*P* ≥ 0.05, *r* = 0.618, 0.600, 0.806, 0.706, and 0.747, respectively). Significantly positive (*P* < 0.05) correlations were detected between SUA and the abundance of *Sphingobium* (*r* = 0.915), and between soil URA and the abundance of the bacterium *Priestia* (*r* = 0.918). In contrast, soil URA and SUA were significantly negatively correlated (*P* < 0.05 or *P* < 0.01, *r* = −0.918, −0.937) with the abundance of the bacterium *Rhizobium* (Figure 7A and Supplementary Figure 10). Abundances of the fungal genera *Hannaella*, *Verticillium*, *Bisifusarium*, and *Vishniacozyma* were significantly positively (*P* < 0.05 or *P* < 0.01) correlated with soil OM concentration, but were significantly negatively (*P* < 0.05) correlated with soil AN concentration. The abundances of the fungal genera *Bulleromyces* and *Boothiomyces* were significantly positively (*P* < 0.05 or *P* < 0.01) correlated with soil PHA. Significantly positive (*P* < 0.05) correlations were detected between the abundances of the genera *Acrocalymma* and *Sporobolomyces* and URA. In addition, soil AK concentration was significantly positively (*P* < 0.05) correlated with the abundances of the *Lectera*, *Trechispora*, *Idriella*, and *Papiliotrema* genera (Figure 7B and Supplementary Figure 11). The results implied that soil properties also were closely related to the composition of microbial communities.

Naturally, microorganisms live in complicated ecological networks (Shi et al., 2022; Zengler and Zaramela, 2018). Some microbes living in complicated network environment commonly depend on neighbor microorganism to obtain nutrients or bio-signal, which may be beneficial for them to grow and multiply in environment (D’Onofrio et al., 2010). Certainly, competitive or antagonistic interactions also exist among microbes (Yan et al., 2023). In current study, both synergistic (positive) and antagonistic (negative) interactions were detected among the microbes which displayed significant positive and negative relationships with some agronomic traits of LLP (Supplementary Figure 12). The finding gives cues for biocontrol strategy of phytopathogen or application of LLP growth-promoting microbes as friendly bio-fertilizers, which will be reported in forthcoming works.

### 4.3 Niche differences in terms of microbial community diversity and structure

Numerous microbial communities and their plant hosts form “holo-bionts,” assemblages of the microorganism species living around or within the host (Aziz et al., 2022). Interplay between the microbiota and the host plant improves anti-pathogen defense, nutrient uptake, and plant growth (Trivedi et al., 2020). The health state and organ of the host plants can affect species richness and diversity of the microbes interacting with the host (Shade et al., 2013; Almario et al., 2022). In the present study, the α-diversity indices of the species richness (ACE) and diversity (Shannon) demonstrated dynamic fluctuations among the RS and different vegetative organs of the LLP plants. Bacterial species richness and diversity in the two health states of LLPs were highest in the RS, significantly (*P* < 0.05) higher than those of organ samples, and with no significant differences among the stem and leaf samples and between DRS and HRS samples (Figure 5A, B). Increasing microbes in RS is beneficial for plants absorbing nutrients and resisting diseases, inversely, the root systems of plants also provide living space and nutrients (e.g., saccharides, amino acid, organic acid and others). The symbiotic interplays further contribute to the increasing in bacterial diversity and richness (Hacquard et al., 2015; Yamamoto et al., 2018). In previous works, Cai et al. (2025) reported that the Shannon index of bacteria was not significantly different among the roots, stems and leaves of five phytophthora-resistant pepper cultivars leaves, generally, ACE index was the highest in leave, but it not mentioned microbial diversity in RS. Dai et al. (2019) reported that bacterial ACE and Shannon in rhizosphere were significantly different between healthy and blight disease-affected peppers, while it did not mention bacterial diversity of the pepper organs, which was not consist with the current study, it maybe because of different pathogen, field environment and others. In contrast, the species richness and diversity of fungi were not significantly different among the various organs, except for the Shannon index in the DS and HRS, which was significantly higher than that in the HS (Figure 5C, D). These findings did not agree with those of a previous study in which fungal richness was highest in roots and lowest in stems of the medicinal plant *Cynanchum bungei* (Chen et al., 2020), possibly because of differences in stress conditions, such as pathogens, nutrients, and drought. The bacterial and fungal β-diversities were significantly different in all samples. The PCoA results indicated that three clusters (RS; root; stem and leaf were a cluster because of the near Bray–Curtis distance from each other) could be separated individually by the bacteria PCoA1 and the fungal PCoA2 in terms of community structure (Figure 4A, B). Previous work had also reported that endophytic bacterial communities of pepper fruits and roots clustered separately based on non-metric multidimensional scaling (NMDS) analysis (Cui et al., 2020).

A shift in the bacterial and fungal community structure occurred in response to different health state niches. 11 bacterial phyla with a relative abundance greater than 1% were used to construct bar charts, and the six bacterial phyla Pseudomonadota, Acidobacteria, Bacillota, Actinomycetota, Proteobacteria, and Bacteroidota constituted more than 58.09% of the total bacteria in the RS and pepper organs in the two health states. Although there was no significant difference (*P* ≥ 0.05) in the abundance of Pseudomonadota among the stem and leaf samples from diseased and healthy pepper plants, its abundance in healthy roots was significantly higher (*P* ≥ 0.05) than that in diseased roots, suggesting that this bacterial phylum in roots might protect plants from pathogens and promote fitness and development of the pepper plants. In contrast, for Bacillota, the bacterial phylum abundances in DR and DS samples were significantly higher (*P* < 0.05) than other samples (Figure 4C and Supplementary Table 13). It suggested that bacterial community structure at the phylum level was different with different health state and niches, which was consistent with the study of Cai et al. (2025) in niche variation of bacterial phyla. For fungi, five phyla, the Ascomycota, Basidiomycota, Mortierellomycota, Chytridiomycota, and Glomeromycotamade, with abundances above 1%, were used to construct a bar chart; the abundances of these phyla accounted for more than 89% of the total fungi in all samples. The abundance of the fungal phylum Ascomycota ranged from 41.36% to 68.50%, and it gradually increased in diseased pepper plants and decreased in healthy pepper plants with variations in the vegetative organs. However, the fungal phylum Basidiomycota exhibited the opposite trend, indicating that the different communities varied widely (Figure 4D and Supplementary Table 15). The top four fungal phyla were same as resistant *Phytophthora* blight (SJ-0948 and CM334) and Susceptible *Phytophthora* blight (Fudijian and Qiemen) pepper cultivars, but the trends in the abundances fluctuation of the phylum fungi were different (Cai et al., 2025), it might be because of different pepper cultivars, pathogens, and environments.

### 4.4 Effects of plant disease on pepper agronomic performance

Pepper pathogens can impact plant growth and health in severe cases, leading to a large decrease in crop productivity (Ramos-Garcia et al., 2016; Cheon et al., 2015; Utkhede and Mathur, 2003). For instance, chili damping-off and blight, which are individually caused by pathogenic fungi *Pythium aphanidermatum* and *Phytophthora capsica*, lead to pepper collapse or withering rapidly (Shen et al., 2023). In the present study, LLP disease was possibly caused by several fungal and bacterial pathogen communities, as described above; the potential pathogens were speculated via 16S and ITS high-throughput sequencing and alignment with SILVA and UNITE databases. The results indicated that plant height, fresh weight, and chlorophyll a significantly (*P* < 0.05) decreased in diseased plants and that POD, SOD activity and proline concentration increased in diseased plants compared with the levels in healthy pepper plants, suggesting that disease-related oxidative stress impacts pepper growth and fitness (Figure 1). Additionally, pepper fruit quality was also affected by disease stress. Compared with the fruits on healthy pepper plants, the fruit fresh weight and Vc concentration of diseased LLP plants decreased significantly (*P* < 0.05) (Figure 2). However, unexpected trends were presented in current experiment, compared with LLP healthy fruits, higher soluble sugar content, more red fruits (Figures 2C, E), and lower MDA concentrations (Figure 1E) were detected in diseased fruits and leaves, respectively. Pepper infected with pathogens exhibited abnormal physiological function. For example, due to the obstruction of photosynthetic product transport, sugar might accumulate abnormally in fruits, and negatively related to MDA concentrations. Meanwhile, disease accelerated fruit ripening prematurely. Peppers might enhance their osmotic regulation abilities by increasing the concentration of soluble sugar, improving the protein’s water-binding ability and better maintaining the permeability of the cell membrane to alleviate the denaturation of enzyme protein caused by osmic stress, and there playing a role resist pathogen. The increasing in sugar or decreasing in MDA concentrations were usually temporary. In the long term, the disease will eventually lead to a decline in the ability of sugar synthesis and resistance (Peng et al., 2016; Baba et al., 2019; Gao and Xie, 2021; Li et al., 2006).

### 4.5 Validation of fungal pathogens YW25 and YW28 on pepper plants

To validate the LLP disease caused by pathogens, two fungal isolates *Fusarium* genus YW25 (*F. proliferatum*) and YW28 (*F. oxysporum*) were isolated and incubated to the pepper roots. Although the genus, diverse and global distribution, is associated with plants, animal, and human as both pathogenic and non-pathogenic species, the disease of pepper plant infected by *F. oxysporum* is a devasting disease leading to severe vegetable losses (Mariyappa et al., 2025), and is definite pepper-wilt (Zhang et al., 2022; Zhang et al., 2021). Additionally, the pathogen genus *F. proliferatum* was reported to cause leaf blight disease of pepper plant in India (Saha and Tayung, 2025), but in our validation experiment, this pathogen also led to the yellow leaves of pepper seedlings. The choice of root incubation method was based on its high relative abundance in roots of diseased LLP pants in field investigation, which was significant higher (*P* < 0.05) in the DR sample than the other samples (DS, DL, DRS, HR, HS, HL, and HRS) (Supplementary Figure 8), additionally, previous studies were referred (Li et al., 2023; Liu, 2023). In our validation experiment, after inoculation with YW25 and YW28 for 7–10 days, the leaves of pepper seedlings obviously become yellow contrast to CK (Figures 8A–C), which was consistent with previous reports on soybean (Liu, 2023), chili pepper (Majeed et al., 2024), and banana plant (Chen et al., 2024). However, the symptom of pepper leaf infected by *F. proliferatum* was not consistent with Saha and Tayung’s (2025) study in India, which might be because of different *F. proliferatum* races and environments, it also provided the new idea of pepper-wilt pathogen research and control in the future. When these two pathogens colonized in the pepper roots, they might restrict plants to uptake nutrients or inhibited the growth of saprophytic microbes in rhizosphere soil, the leaves of pepper turned yellow due to a lack of nutrients (Abo-Elyousr et al., 2024). With the development of molecular biotechnology, RT-qPCR is used to determine the relative biomass of pathogen in infected plants by special primer pair designed (Qiao et al., 2025; Varela et al., 2025). In our experiment, the results of RT-qPCR analysis showed that both pathogens YW25 and YW28 colonized in the pepper plants and reproduced in large numbers (Table 2).

Nevertheless, the current work is not without its weakness. Firstly, based on the high-throughput sequencing results in limited time, we concentrated to align and speculate the possible pathogens of the LLP. Experimental validation of the pepper pathogens was conducted, and two fungal pathogens were isolated, there might be other culturable pathogens causing LLP disease via Koch’s postulate, which will be reported in our forthcoming studies. There might also be un-cultured pathogens causing LLP disease because 1% microbes could be isolated and cultured on nutrient medium, which is expected to be solved using more advanced molecular technique. Secondly, as for early prediction and real-time monitoring of the wilt disease in field pepper, RT-qPCR was just applied to analyze the relative biomass of two pathogen strains in infected pepper seedlings in this study, integrating pathogenic molecular data with pepper physiological and environmental data requires extensive and systematic exploration, which is one of our forthcoming research aims. Thirdly, the investigation in the same field was conducted using small sample size (nine diseased replicates, with three plants per replicate, a total of nine diseased LLP, healthy LLP collection was same as diseased LLP), the main reason was resource limitations. Fourthly, our study did not perform meta-transcriptomic validation or functional assays to confirm microbial activity. All in all, we will overcome these limitations and report our more complete studies in the future.

## 5 Conclusion

In the present study, the plant growth and fruit quality of LLP plants were impacted by disease stress. Following the Koch’s rule, two candidates of pepper-wilt pathogenic YW25 and YW28 were purified and identified, which provided the proof for correctness of our work. Moreover, there were niche differences exhibited by some microbial communities in different plant organs and the RS between healthy and diseased plants, and the recruitment of pepper for microbes was dissimilar to difference of the health status of pepper plants. Correlation analyses revealed that the abundance of some members of the microbial communities were significantly positively or negatively related to LLP growth, physiology, and fruit quality in response to disease challenge, suggesting that some microbes play important roles in pepper growth and fitness, while other microbes might inhibit pathogen development in peppers. However, the mechanisms, by which interactions among potential beneficial microbes and host LLP plants occur, resulting in pathogen resistance, need to be further explored.

## Data Availability

The original contributions presented in the study are publicly vailable in the NCBI repository. The accession number ID is PRJNA1306756, This data can be found here: http://www.ncbi.nlm.nih.gov/bioproject/1306756.

## Footnotes

1
http://www.arb-silva.de/

2
https://unite.ut.ee/index.php

3
https://www.omicshare.com/tools/Home/Soft/pcoa

4
https://www.omicshare.com/tools

5
https://blast.ncbi.nlm.nih.gov/Blast.cgi
