# Supplementary material for: Microbial niche differentiation and agronomic performance of diseased Capsicum annuum
Source: Front Microbiol. 2025 Sep 3;16:1576486. doi: 10.3389/fmicb.2025.1576486 (PMC12440944; doi:10.3389/fmicb.2025.1576486)
Supplement: Supplementary file 1 [file Data_Sheet_1.pdf]

Microbial Niche Differentiation and Agronomic Performance of Diseased *Capsicum annuum*

Zhiqi Yang[1], and Yankun Wang[1,2,*]

1. College of Agriculture, Fujian Agriculture and Forestry University, Fuzhou, Fujian, China, 350000;

2. College of Bee Science and Biomedicine, Fujian Agriculture and Forestry University, Fuzhou, Fujian, 350000)

* Correspondence: Yankun Wang, E-mail： fafuwyk@163.com

Supplementary tables and figures

DNA extract and amplification

Details or justification for choosing the specified primers for V3–V4 and ITS2 regions.

16S sequencing is to amplify and sequence the target fragment region (that is a part of 16S rDNA) of the total DNA extracted from the sample, and after analysis of sequencing data, the microbial diversity and community structure in the sample. 16S rDNA is composed of 10 conserved regions and 9 variable regions. The conserved regions can be used to judge the genetic relationship among species, while the variable region sequence can be used to explore the difference among species. Due to the read length limitation of second-generation sequencing platform, only single V region, double V region or triple V region can be selected as the target fragment region for 16S sequencing. Since the good specificity of V3, V4, and V5, and the relatively complete database information. The V3–V4, V4 or V4–V5 are often selected for bacterial diversity annotation in second-generation sequencing. Among them, primers 341F and 806R are selected for V3–V4 amplification, which have a high coverage rate in bacterial and could detect the diversity distribution of bacteria. In our experiment, the V3–V4 variable region bands of 16S rRNA gene amplified by primers 341F and 806R were uniform and clear (Supplementary Figure 2), which could be used for subsequent tests after recovery.

The ITS sequence is a very useful identification tool for fungi because it is a part of the ribosomal RNA gene cluster of smaller subunits in fungi, which is an essential part of RNA synthesis in eukaryotes. The ITS sequence refers to the DNA sequence containing the transcription spacer 1 (ITS1) and 2 (ITS2) within the ribosomal RNA gene cluster, which are located at the 5' and 3' ends of the ribosomal RNA gene, respectively. The ITS sequence is a small, highly variable gene region that is often used to identify a specific species because it is relatively conserved and variable at the specie level, which makes it easier to compare among different species. In our analysis, the ITS rRNA gene amplified by primers ITS1F and ITS2R had uniform and clear ribbons ITS variable region (Supplementary Figure 3), which could be used for subsequent fungal sequencing tests after recovery.

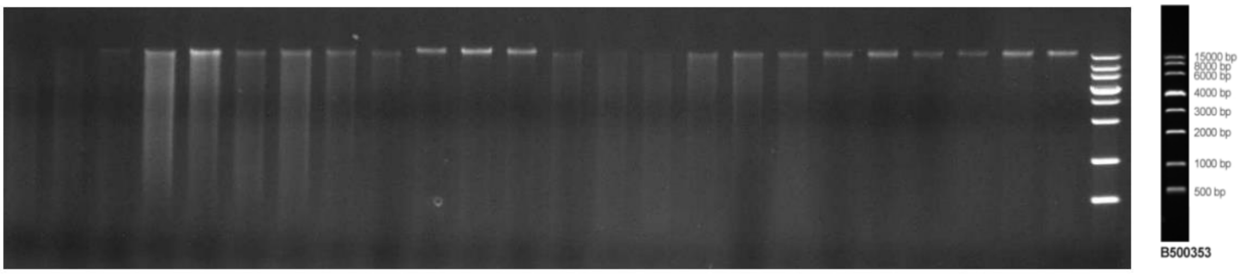

Supplementary Figure 1 Electrophoretogram of total DNA abstracted from 24 sub-samples. The order of sample dots from left to right: DR-1, DR-2, DR-3, DS-1, DS-2, DS-3, DL-1, DL-2, DL-3, DRS-1, DRS-2, DRS-3, HR-1, HR-2, HR-3, HS-1, HS-2, HS-3, HL-1, HL-2, HL-3, HRS-1, HRS-2, and HRS-3.

Supplementary Table 1 The concentrations of total DNA abstracted from 24 sub-samples.

Sample ID	Concentration (ng/μL)	Sample ID	Concentration (ng/μL)	Sample ID	Concentration (ng/μL)
DR-1	18.78	DR-2	15.37	DR-3	8.46
DS-1	81.47	DS-2	54.29	DS-3	30.25
DL-1	46.03	DL-2	25.14	DL-3	12.86
DRS-1	11.51	DRS-2	17.31	DRS-3	10.4
HR-1	12.66	HR-2	13.43	DRS-3	8.93
HS-11	13.72	HS-2	22.42	HS-3	16.14
HL-1	5.85	HL-2	9.25	HL-3	6.16
HRS-1	4.7	HRS-1	12.3	HRS	9.22

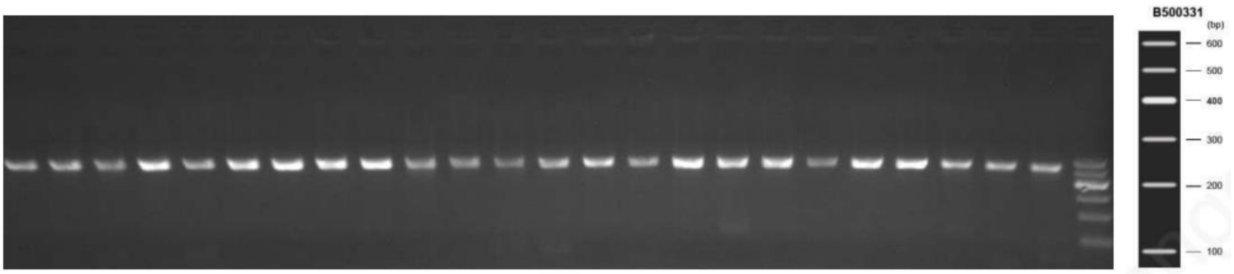

Supplementary Figure 2 Amplification of bacterial 16S rRNA gene from the total DNA of 24 sub-samples. The order of sample dots from left to right: DR-1, DR-2, DR-3, DS-1, DS-2, DS-3, DL-1, DL-2, DL-3, DRS-1, DRS-2, DRS-3, HR-1, HR-2, HR-3, HS-1, HS-2, HS-3, HL-1, HL-2, HL-3, HRS-1, HRS-2, and HRS-3. In current experiment, the V3-V4 variable region bands, which were amplified by primers 341F (5′-CCTACGGGNGGCWGCAG-3′) and 806R (5′-GACTACHVGGGTATCTAATCC-3′) for V3–V4, were uniform and clear, the amplification product could be used for follow-up sequencing tests after recovery.

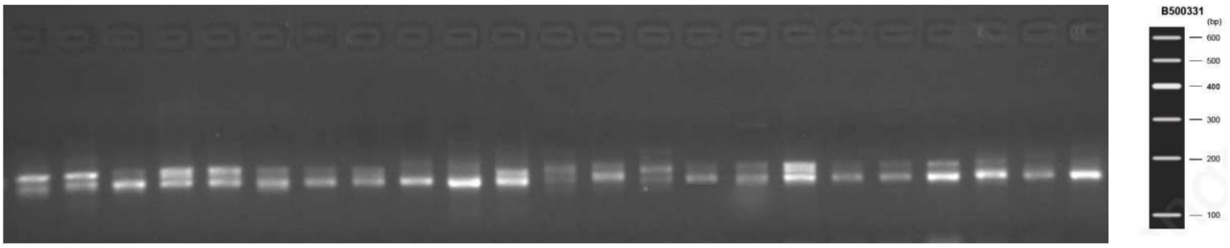

Supplementary Figure 3 Amplification of fungal ITS rRNA gene from the total DNA from 24 sub-samples. The order of sample dots from left to right: DR-1, DR-2, DR-3, DS-1, DS-2, DS-3, DL-1, DL-2, DL-3, DRS-1, DRS-2, DRS-3, HR-1, HR-2, HR-3, HS-1, HS-2, HS-3, HL-1, HL-2, HL-3, HRS-1, HRS-2, and HRS-3. In current experiment, the ITS region bands of ITS rRNA gene, which were amplified by primers ITS1F 5′-CTTGGTCATTTAGAGGAAGTAA-3′ and ITS2R 5′-GCTGCGTT CTTCATCGATGC-3′ for ITS regions, were uniform and clear, the amplification product could be used for follow-up sequencing tests after recovery.

PCR amplification mixture

Supplementary Table 2 The composition of the PCR reaction mixture in the first and second amplifications of 24 sub-samples.

PCR amplification	Component	Volume	Total volume
The first amplification	2×Hieff® Robust PCR Master Mix (polymerase, Yeasen, 10105ESO3)	15 μL	30 μL
	Bar-PCR primer F	1 μL	
	Primer R	1 μL	
	PCR products	10–20 ng	
	H_2O	9–12 μL	
The second amplification	2×Hieff® Robust PCR Master Mix (polymerase, Yeasen, 10105ESO3)	15 μL	30 μL
	Primer F	1 μL	
	Index-PCR Primer R	1 μL	
	PCR products	20–30 ng	
	H_2O	9–12 μL	

Note: For the first amplification, the bacterial (16S) primer pair was 341F (Bar-PCR primer F) 5′-CCTACGGGNGGCWGCAG-3′ and 805R (Primer R) 5′-GACTACHVGGGTATCTAATCC-3′; The Fungal (ITS) primer pair was ITS1F (Bar-PCR primer F) 5′-CTTGGTCATTTAGAGGAAGTAA-3′ and ITS2R (Primer R) 5′-GCTGCGTT CTTCATCGATGC-3′. For the second amplification, the adapters were P5 (Primer F) 5′-AATGATACGGCGAC CACCGAGATCTACAC-3′ and P7 (Index-PCR Primer R) 5′-CAAGCAGAAGACGGCATACGAGAT-3′, and same in 16S and ITS PCR amplification.

The PCR reaction mixture and ITS sequences of isolated fungal pathogenic DNA

Supplementary Table 3 The composition of the PCR reaction mixture in the amplifications of isolated fungal pathogenic DNA.

Component	Volume	Total volume
Template (extracted DNA of fungal pathogen)	1 μL	
Primer up (ITS1, 10 μmol L^{-1})	0.5 μL	
Primer down (ITS4, 10 μmol L^{-1})	0.5 μL	20 μL
dNTP mix (10 mmol L^{-1} each)	0.5 μL	
10 x Taq reaction Buffer	2.5 μL	
Taq polymerase	0.2 μL	
H_2O	14.8 μL	

Note: The common primer pair ITS1(5'-TCCGTAGGTGAACCTGCGG-3') and ITS4 (5'-TCCTCCGCTTATTGATATGC-3') were applied to amplify ITS V1-V2 region of fungal rDNA, and the PCR product length was 600 bp.

Supplementary Table 4 The sequences of fungal pathogens YW25 *Fusarium proliferatum* and YW28 *Fusarium Oxysporum*.

	**Gene sequence**
YW25	GCCTGCGGAGGGATCATTACCGAGTTTACAACTCCCAAACCCCTGTGAACATACCAATTGTTGCCTCGGCGGATCAGCCCGCTC<br>CCGGTAAAACGGGACGGCCCGCCAGAGGACCCCTAAACTCTGTTTCTATATGTAACTTCTGAGTAAAACCATAAATAAATCAAA<br>ACTTTCAACAACGGATCTCTTGGTTCTGGCATCGATGAAGAACGCAGCAAAATGCGATAAGTAATGTGAATTGCAGAATTCAGT<br>GAATCATCGAATCTTTGAACGCACATTGCGCCCGCCAGTATTCTGGCGGGCATGCCTGTTCGAGCGTCATTTCAACCCTCAAGCC<br>CTCAGGTTTGGTGTTGGGGATCGGCGAGCCCTTGCGGCAAGCCGGCCCCGAAATCTAGTGGCGGTCTCGCTGCAGCTTCCATTG<br>CGTAGTAGTAAAACCCTCGCAACTGGTACGCGGCGCGGCCAAGCCGTTAAACCCCCAACTTCTGAATGTTGACCTCGGATCAG<br>GTAGGAATACCCGCTGAACTTAAGCATATCAAAT
YW28	TTCCGTAAGGGGGGCCTGCGGAGGGATCATTACCGAGTTTACAACTCCCAAACCCCTGTGACATACCAATTGTTGCCTCGGCGG<br>ATCAGCCCGCTCCCGGTAAAACGGGACGGCCCGCCAGAGGACCCCTAAACTCTGTTTCTATATGTAACTTCTGAGTAAAACCAT<br>AAATAAATCAAAACTTTCAACAACGGATCTCTTGGTTCTGGCATCGATGAAGAACGCAGCAAAATGCGATAAGTAATGTGAATT<br>GCAGAATTCAGTGAATCATCGAATCTTTGAACGCACATTGCGCCCGCCAGTATTCTGGCGGGCATGCCTGTTCGAGCGTCATTTC<br>AACCCTCAAGCCCTCAGGTTTGGTGTTGGGGATCGGCGAGCCCTTGCGGCAAGCCGGCCCCGAAATCTAGTGGCGGTCTCGCT<br>GCAGCTTCCATTGCGTAGTAGTAAAACCCTCGCAACTGGTACGCGGCGCGGCCAAGCCGTTAAACCCCCAACTTCTGAATGTTG<br>ACCTCGGATCAGGTAGGAATACCCGCTGAACTTAAGCATATCAAA

RT-qPCR analysis of fungal pathogenic DNA from infected pepper seeding roots.

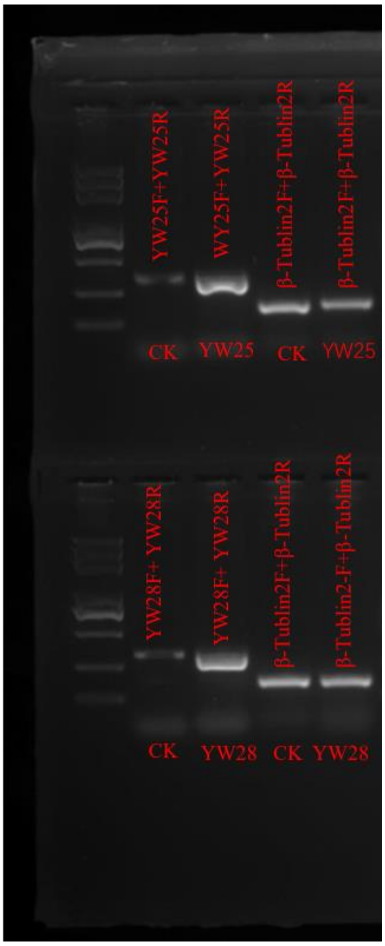

Supplementary Figure 4 Representative agarose gel electrophoresis of abstracted specific DNA of both fungal pathogens from pepper seedling roots treated by YW25 and YW28, respectively.

YW25/28F, 5′-ACTCCCAAACCCCTGTGAAC-3;

YW25/28R, 5′-CACCAAACCTGAGGGCTTGA-3.

*Tublin*2F, 5′-GAGGGTGAGTGAGCAGTTC-3;

*Tublin*2R, 5′-CTTCATCGTCATCTGCTGTC-3.

Supplementary Table 5 The primers of the RT-qPCR reaction in the amplifications of fungal pathogenic DNA from infected pepper seeding roots.

primers	Sequences	Product size (bp)	Type
qRJD25/28F	5′-GGCATCGATGAAGAACGCAG-3'	162	ITS
qRJD25/28R	5′-TCCCCAACACCAAACCTGAG-3'		
*β-Tublin*2-F	5′-GAGGGTGAGTGAGCAGTTC-3'	About 150	internal standard
*β-Tublin*2-R	5′-CTTCATCGTCATCTGCTGTC-3'		

Supplementary Table 6 The composition of the RT-qPCR reaction mixture in the amplifications of fungal pathogenic DNA from infected pepper seeding roots.

Component	Volume	Total volume
ChamQ SYBR Green PCR master Mix	10 µL	
RT-F primer	0.5 µL	
RT-R primer	0.5 µL	20 µL
cDNA	2 µL	
ddH_2O	7 µL	

Note: Because both YW25 and YW28 belong to a same genus *Fusarium*, the primer pair qRYW25/28F (5'-GGCATCGATGAAGAACGCAG-3') and qRYW25/28R (5'-TCCCCAACACCAAACCTGAG-3') were used in the RT-qPCR reaction. ChamQ SYBR Green PCR master Mix included buffer, dNTP, Taq DNA polymerase, SYBR Green I dye, and $MgCl_2$.

Supplementary Table 7 The raw reads of bacterial communities in long line pepper (LLP) samples.

Group	Sample	Barcode	SeqNum	BaseNum	MeanLen	MinLen	MaxLen
DR	DR-1	CGCCAT	38661	17460051	451.62	39	482
DR	DR-2	GAGGTT	47635	21473078	450.78	43	482
DR	DR-3	GCCGCT	53618	24278548	452.81	42	497
DS	DS-1	GAAACC	41165	18256178	443.49	41	482
DS	DS-2	CACGAT	40789	18108783	443.96	40	485
DS	DS-3	GCGGTA	43232	19178618	443.62	39	478
DL	DL-1	TATCGA	46645	20689381	443.55	43	482
DL	DL-2	CGCATA	42412	18806879	443.43	42	475
DL	DL-3	CTTGTA	42231	18604209	440.53	41	471
DRS	DRS-1	GTTTCG	49080	22045059	449.17	40	505
DRS	DRS-2	CTCCTG	50184	22579566	449.94	39	502
DRS	DRS-3	GTCGGA	63038	28357409	449.85	43	506
HR	HR-1	ATCGTT	37292	16542111	443.58	42	491
HR	HR-2	AATATC	43323	19245311	444.23	41	472
HR	HR-3	AAGCTC	37041	16447043	444.02	40	486
HS	HS-1	TTCCAT	43802	19348043	441.72	39	475
HS	HS-2	TCTAGG	46024	20096138	436.64	43	487
HS	HS-3	CTATAC	39723	17614656	443.44	42	475
HL	HL-1	GTCCCA	39729	17633638	443.85	42	470
HL	HL-2	ATCGCA	38299	16991507	443.65	40	480
HL	HL-3	TTACGA	39594	17561799	443.55	39	470
HRS	HRS-1	TGTTAT	57415	25694750	447.53	43	504
HRS	HRS-2	GCCATC	51088	22881123	447.88	42	510
HRS	HRS-3	TGTGTT	52872	23565384	445.71	41	504
Sum			1084892				

Note: DRS represents rhizosphere soil from diseased LLP plant; DL, DS and DR represent the leaf, stem and root of diseased LLP plant, respectively. HRS shows rhizosphere soil from healthy LLP plant; HL, HS and HR individually represent the leaf, stem and root of healthy LLP plant.

Supplementary Table 8 The clean reads of bacterial communities in long line pepper (LLP) samples.

Group	Sample	Barcode	SeqNum	BaseNum	MeanLen	MinLen	MaxLen	OTUs classified
DR	DR-1	CGCCAT	35997	14958232	415.54	351	444	292
DR	DR-2	GAGGTT	44608	18435404	413.28	351	444	306
DR	DR-3	GCCGCT	50224	20832453	414.79	350	459	282
DS	DS-1	GAAACC	38680	15700923	405.92	350	444	406
DS	DS-2	CACGAT	38404	15590335	405.96	350	447	645
DS	DS-3	GCGGTA	40497	16445316	406.09	350	440	754
DL	DL-1	TATCGA	43752	17754438	405.8	350	444	1542
DL	DL-2	CGCATA	39720	16120296	405.85	350	437	1662
DL	DL-3	CTTGTA	39259	15937493	405.96	350	432	1503
DRS	DRS-1	GTTTCG	45469	18833656	414.21	352	467	301
DRS	DRS-2	CTCCTG	46521	19288510	414.62	351	464	264
DRS	DRS-3	GTCGGA	58594	24277784	414.34	350	468	299
HR	HR-1	ATCGTT	35030	14228568	406.18	351	453	250
HR	HR-2	AATATC	40456	16497014	407.78	350	434	227
HR	HR-3	AAGCTC	34793	14144978	406.55	351	448	239
HS	HS-1	TTCCAT	41079	16671355	405.84	350	437	453
HS	HS-2	TCTAGG	42609	17289040	405.76	350	449	486
HS	HS-3	CTATAC	37283	15134849	405.95	350	437	499
HL	HL-1	GTCCCA	37269	15127583	405.9	353	432	1574
HL	HL-2	ATCGCA	35907	14574024	405.88	351	442	1426
HL	HL-3	TTACGA	37178	15087500	405.82	351	432	1633
HRS	HRS-1	TGTTAT	53358	21955454	411.47	352	466	275
HRS	HRS-2	GCCATC	47425	19517259	411.54	350	472	300
HRS	HRS-3	TGTGTT	48690	20086522	412.54	350	464	302
Sum			1012802					15920

Note: DRS represents rhizosphere soil from diseased LLP plant; DL, DS and DR represent the leaf, stem and root of diseased LLP plant, respectively. HRS shows rhizosphere soil from healthy LLP plant; HL, HS and HR individually represent the leaf, stem and root of healthy LLP plant.

Supplementary Table 9 The raw reads of fungal communities in long line pepper (LLP) samples.

Group	Sample	Barcode	SeqNum	BaseNum	MeanLen	MinLen	MaxLen
DR	DR-1	ACGAGTG	61538	17386665	282.54	48	493
DR	DR-2	ACGCTCG	58961	16825853	285.37	42	492
DR	DR-3	AGACGCA	68305	20065109	293.76	42	480
DS	DS-1	AGCACTG	81044	22045160	272.01	42	488
DS	DS-2	ATCAGAC	79710	24049881	301.72	47	478
DS	DS-3	ATATCGC	63965	19614913	306.65	47	479
DL	DL-1	CGTGTCT	64177	17823542	277.72	44	492
DL	DL-2	CTCGCGT	66131	18362363	277.67	42	492
DL	DL-3	TAGTATC	73138	21262615	290.72	43	488
DRS	DRS-1	TCTCTAT	61669	17315128	280.78	42	490
DRS	DRS-2	TGATACG	74162	20573332	277.41	42	492
DRS	DRS-3	TACTGAG	61099	17910232	293.13	42	492
HR	HR-1	TCACGTG	48360	14407307	297.92	42	492
HR	HR-2	TGCATCT	58736	17240768	293.53	42	492
HR	HR-3	TAGCACA	51501	15355644	298.16	42	492
HS	HS-1	TCGTCGA	54897	16496570	300.5	42	492
HS	HS-2	TGTACTG	63021	17621014	279.61	45	485
HS	HS-3	TCAGTAC	68854	20839306	302.66	42	479
HL	HL-1	TATAGCA	69255	19750134	285.18	43	488
HL	HL-2	TGCTATG	55790	15519137	278.17	42	492
HL	HL-3	TCTAGTC	65781	18327315	278.61	42	492
HRS	HRS-1	TACGACA	59651	16746027	280.73	45	488
HRS	HRS-2	TGTGCTA	65055	18906141	290.62	47	490
HRS	HRS-3	TCACTCG	74307	20507926	275.99	42	492
Sum			1549107				

Note: DRS represents rhizosphere soil from diseased LLP plant; DL, DS and DR represent the leaf, stem and root of diseased LLP plant, respectively. HRS shows rhizosphere soil from healthy LLP plant; HL, HS and HR individually represent the leaf, stem and root of healthy LLP plant.

Supplementary Table 10 The clean reads of fungal communities in long line pepper (LLP) samples.

Group	Sample	Barcode	SeqNum	BaseNum	MeanLen	MinLen	MaxLen	OTUs classified
DR	DR-1	ACGAGTG	61376	14784265	240.88	106	451	466
DR	DR-2	ACGCTCG	58736	14322030	243.84	103	450	482
DR	DR-3	AGACGCA	68238	17186555	251.86	113	438	403
DS	DS-1	AGCACTG	80466	18627470	231.49	100	446	430
DS	DS-2	ATCAGAC	79622	20692143	259.88	102	436	383
DS	DS-3	ATATCGC	63836	16915293	264.98	103	437	403
DL	DL-1	CGTGTCT	64034	15116346	236.07	100	450	494
DL	DL-2	CTCGCGT	65798	15558304	236.46	100	450	548
DL	DL-3	TAGTATC	73010	18175907	248.95	100	446	504
DRS	DRS-1	TCTCTAT	61533	14677177	238.53	101	443	538
DRS	DRS-2	TGATACG	73974	17403180	235.26	105	450	397
DRS	DRS-3	TACTGAG	60999	15310394	250.99	101	450	399
HR	HR-1	TCACGTG	48037	12345067	256.99	100	450	180
HR	HR-2	TGCATCT	58568	14749484	251.84	100	450	370
HR	HR-3	TAGCACA	51239	13153240	256.7	107	450	477
HS	HS-1	TCGTCGA	54599	14164220	259.42	100	450	388
HS	HS-2	TGTACTG	62872	14966225	238.04	106	443	406
HS	HS-3	TCAGTAC	68772	17941717	260.89	101	437	443
HL	HL-1	TATAGCA	69211	16833953	243.23	109	446	475
HL	HL-2	TGCTATG	55700	13169073	236.43	106	450	365
HL	HL-3	TCTAGTC	65708	15557796	236.77	101	450	537
HRS	HRS-1	TACGACA	59588	14216364	238.58	116	446	73
HRS	HRS-2	TGTGCTA	64810	16076925	248.06	142	448	412
HRS	HRS-3	TCACTCG	74128	17358862	234.17	100	450	334
Sum			1544854					9907

Note: DRS represents rhizosphere soil from diseased LLP plant; DL, DS and DR represent the leaf, stem and root of diseased LLP plant, respectively. HRS shows rhizosphere soil from healthy LLP plant; HL, HS and HR individually represent the leaf, stem and root of healthy LLP plant.

The rarefaction curves of Shannon indexes for bacteria and fungi from rhizosphere and organs of long line pepper.

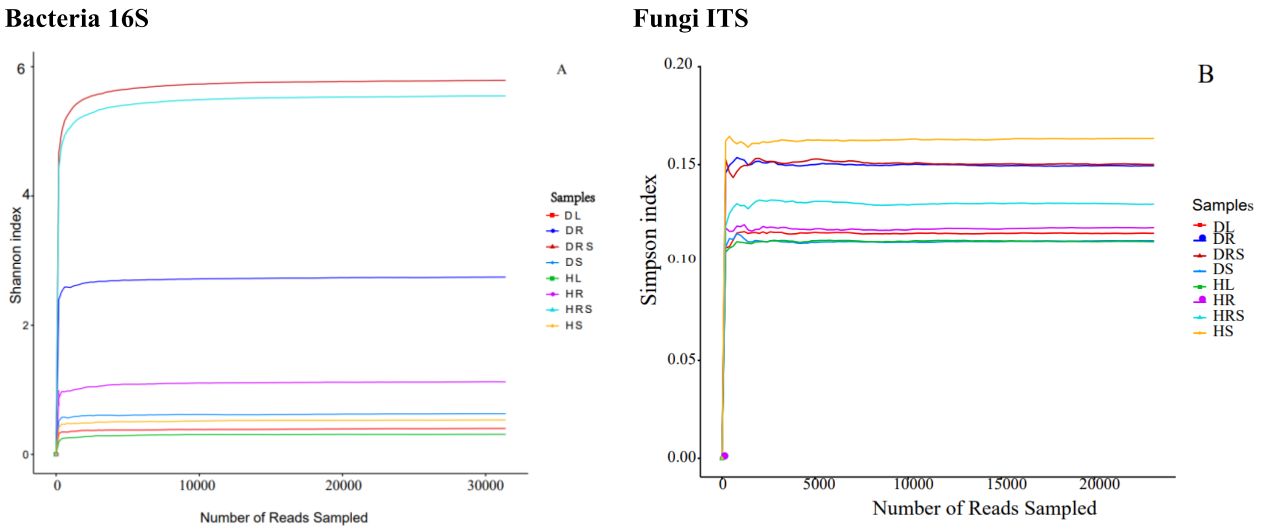

Supplementary Figure 5 The rarefaction curves of Shannon indexes for bacteria (**A**) and fungi (**B**)from rhizosphere soil (RS) and organs of long line peppers (LLPs). A and B represent rarefaction curves of Shannon indexes for bacterial 16S and fungal ITS, respectively.

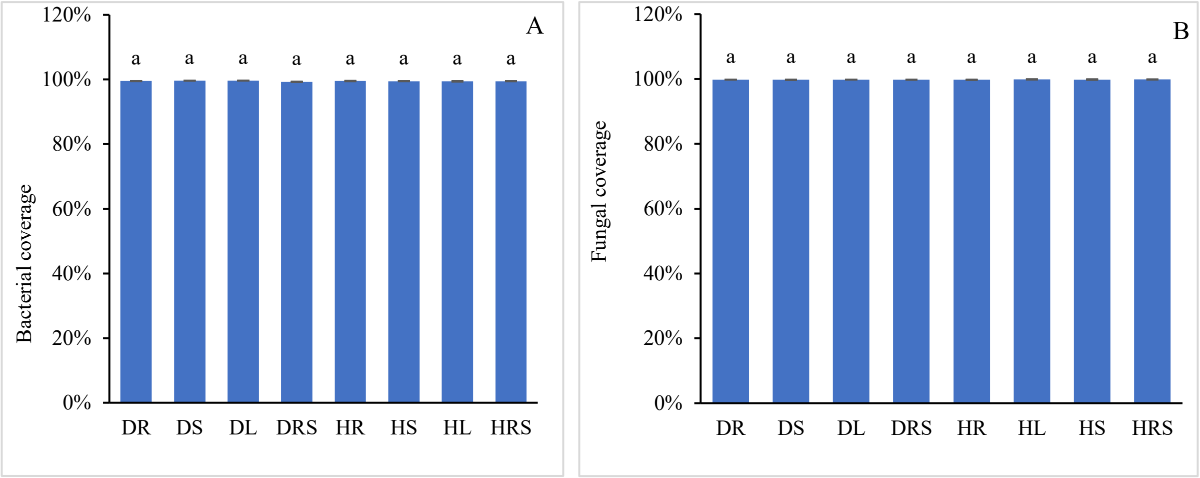

Supplementary Figure 6 Library coverage of bacteria and fungi from rhizosphere soil (RS) and organs of long line pepper (LLP) plants. **A** and **B** represent bacterial and fungal coverage. By SPSS software, after assumptions of normality and homogeneity of variances had been tested using Shapiro-Wilk and Levene' methods, respectively, multiple comparation was conducted using ANOVA and post hoc Tamhane test

Permutational multivariate analysis of variance (PERMANOVA) bacterial and fungal community structure among different pepper niches

Supplementary Table 11 The results of overall PERMANOVA on bacterial community structure from the rhizosphere soil (RS) and organs of long line pepper (LLP) plants in diseased and healthy states, based on OTU data.

Project	Df	SumOSqs	R^2	F	*P*
Group	7	4.501	0.871	15.396	0.001
Residual	16	0.668	0.129	–	–
Total	23	5.170	1.000	–	–

Notes: Df, degrees of freedom, the value is equal to the number of groups compared; SumOSqs, Sums of squares, total variance, also known as the sum of squares of deviation; F, F test value; R^2, variance contribution, represents the interpretation of sample differences in different groups, that is the ratio of group variance to total variance. The larger R^2 is, the higher the interpretation of differences in different groups. *P,* significance *P*-value*,* $P < 0.05$ is a significant difference.

Supplementary Table 12 The results of overall PERMANOVA on fungal community structure from the RS and organs of long line pepper (LLP) plants in diseased and healthy states, based on OTU data.

Project	Df	SumOSqs	R^2	F	*P*
Group	7	4.684	0.687	5.022	0.001
Residual	16	2.132	0.313	–	–
Total	23	6.816	1.000	–	–

Notes: Df, degrees of freedom, the value is equal to the number of groups compared; SumOSqs, Sums of squares, total variance, also known as the sum of squares of deviation; F, F test value; R^2, variance contribution, represents the interpretation of sample differences in different groups, that is the ratio of group variance to total variance. The larger R^2 is, the higher the interpretation of differences in different groups. *P*, significance *P*-value*,* $P < 0.05$ is a significant difference.

The composition of microbial communities at the phylum level from rhizosphere soil (RS) and vegetative organs of long line pepper (LLP) in two health states.

Supplementary Table 13 The relative abundances (%) of bacterial communities at phylum level from rhizosphere soil (RS) and vegetative organs of long line pepper (LLP) in two health states (*n*=3) (means of all samples above 1%).

phylum	DR	DS	DL	DRS	HR	HS	HL	HRS
Pseudomonadota	36.77±4.97b	36.83±7.67b	35.85±5.10b	35.78±1.62b	50.93±4.65a	36.42±4.42b	32.18±4.90b	35.64±2.57b
Acidobacteriota	0.76±0.05d	6.78±1.32c	13.01±2.01b	21.06±1.01a	5.15±0.62c	8.76±1.44c	13.64±2.74b	27.22±4.22a
Bacillota	48.36±8.64a	21.41±0.99b	2.64±0.79c	1.44±0.25c	0.97±0.27d	2.46±0.65c	2.93±0.12c	0.76±0.05d
Actinomycetota	6.53±0.41bc	4.63±0.33c	4.96±0.62c	7.17±1.36b	16.80±3.12a	5.00±0.55c	4.78±0.69c	6.75±0.84b
Patescibacteria	2.00±0.11c	2.85±0.02b	5.16±0.12a	4.20±0.73a	4.77±0.32a	3.27±0.34b	5.18±0.21a	2.42±0.77bc
Bacteroidota	3.67±0.79c	2.02±0.27c	2.47±0.34c	7.28±1.02a	3.62±0.49c	2.16±0.37c	1.55±0.03d	5.38±0.55b
Chloroflexota	0.27±0.06e	1.05±0.40d	2.13±0.35c	7.49±0.40a	0.97±0.23d	2.03±0.25c	1.64±0.26c	3.79±0.52b
Gemmatimonadota	0.09±0.01d	1.37±0.36c	1.84±0.56c	3.96±0.47a	0.90±0.17d	1.74±0.23c	2.89±0.22b	3.86±0.72a
Myxococcota	0.10±0.01d	0.73±0.06c	1.35±0.16b	2.45±0.47a	0.92±0.17c	0.72±0.23c	0.80±0.12c	2.63±0.72a
Planctomycetota	0.06±0.02d	0.42±0.32c	0.67±0.15b	3.16±0.70a	0.32±0.02c	0.70±0.03b	0.80±0.02b	2.97±0.79a
Verrucomicrobiota	0.04±0.00e	0.78±0.07d	1.12±0.05c	2.84±0.25a	0.45±0.05	1.03±0.21cd	1.04±0.23cd	1.64±0.22b
unclassified_Bacteria	1.12±0.40e	19.53±1.90c	25.31±0.85b	0.28±0.02	13.05±2.6d	33.22±3.17a	29.94±0.79a	0.24±0.03f
Others	0.22±0.02e	1.60±0.36d	3.49±0.43b	2.87±0.41bc	1.14±0.33d	2.47±0.21c	2.64±0.25c	6.71±0.42a

Note: DL, DS and DR represent the leaf, stem and root of diseased LLP plant, respectively. DRS and HRS individually show RS from diseased and healthy LLP plant; HL, HS and HR individually represent the leaf, stem and root of healthy LLP plant. Average relative abundances plus standard errors followed by different lowercase letters express significant difference among all samples in row ($P < 0.05$), followed by same lowercase letters express unsignificant difference ($P \geq 0.05$). One-way ANOVA and post hoc by Tamheini test

Supplementary Table 14 The results of overall permutational multivariate analysis of variance (PERMANOVA) on bacterial community composition from the RS and organs of LLP plants in diseased and healthy states, according to their relative abundance data at phylum level.

Project	Df	SumOSqs	R^2	F	*P*
Group	7	1.431	0.787	8.429	0.001
Residual	16	0.388	0.213	–	–
Total	23	1.819	1	–	–

Notes: Df, degrees of freedom, the value is equal to the number of groups compared; SumOSqs, Sums of squares, total variance, also known as the sum of squares of deviation; F, F test value; R^2, variance contribution, represents the interpretation of sample differences in different groups, that is the ratio of group variance to total variance. The larger R^2 is, the higher the interpretation of differences in different groups. *P*, significance P-value, $P < 0.05$ is a significant difference.

Supplementary Table 15 The relative abundances of fungal communities at the phylum level from rhizosphere soil (RS) and vegetative organs of long line pepper (LLP) plants in two health states (n = 3) (means of all samples above 1%).

Fungal phyla	DR	DS	DL	DRS	HR	HS	HL	HRS
Ascomycota	51.65±3.65b	62.40±2.69a	68.50±8.35a	41.58±4.61b	57.86±8.04ab	55.02±5.58ab	49.54±0.55b	41.36±1.13b
Basidiomycota	40.22±0.52a	27.23±3.76b	25.77±6.89b	9.96±3.78c	26.26±7.32b	40.11±4.63a	48.41±1.46a	6.99±2.12c
Mortierellomycota	1.67±0.53c	1.33±0.22c	1.14±0.07c	36.66±6.22a	5.61±1.39b	1.00±0.07c	0.85±0.45c	35.83±4.86a
Chytridiomycota	0.61±0.04c	0.56±0.13c	0.50±0.01c	6.81±1.59a	2.02±0.48b	0.39±0.21c	0.39±0.16c	4.44±1.35a
Glomeromycota	3.73±0.30a	0.07±0.01c	0.06±0.01c	1.13±0.33b	3.62±1.22a	0.06±0.01c	0.04±0.01c	0.61±0.03b
unclassified_Fungi	2.04±0.31c	8.34±1.45a	3.94±0.41b	3.18±0.11b	3.56±0.84b	3.24±0.43b	0.69±0.31d	9.85±0.87a
Others	0.07±0.02b	0.08±0.01b	0.09±0.01b	0.68±0.05a	1.07±0.28a	0.17±0.02b	0.09±0.02b	0.92±0.17a

Note: DL, DS and DR represent the leaf, stem and root of diseased LLP plant, respectively. HRS shows RS from healthy line pepper plant; HL, HS and HR individually represent the leaf, stem and root of healthy LLP plant. The average parameters plus standard errors followed by different lowercase letters express significant difference in same row at $P < 0.05$; followed by same lowercase letters express no significant difference in same row at $P \geq 0.05$. One-way ANOVA and post hoc by Tamheini test

Supplementary Table 16 The results of overall PERMANOVA on fungal community composition from the RS and organs of LLP plants in diseased and healthy states, according to their relative abundance data at phylum level.

Project	Df	SumOSqs	R^2	F	P
Group	7	0.913	0.805	9.433	0.001
Residual	16	0.221	0.211	-	-
Total	23	1.135	1	-	-

Notes: Df, degrees of freedom, the value is equal to the number of groups compared; SumOSqs, Sums of squares, total variance, also known as the sum of squares of deviation; F, F test value; R^2, variance contribution, represents the interpretation of sample differences in different groups, that is the ratio of group variance to total variance. The larger R^2 is, the higher the interpretation of differences in different groups. P, significance P-value, $P < 0.05$ is a significant difference.

Composition heatmaps of bacterial and fungal communities from rhizosphere and organs of long line pepper (LLP) plants.

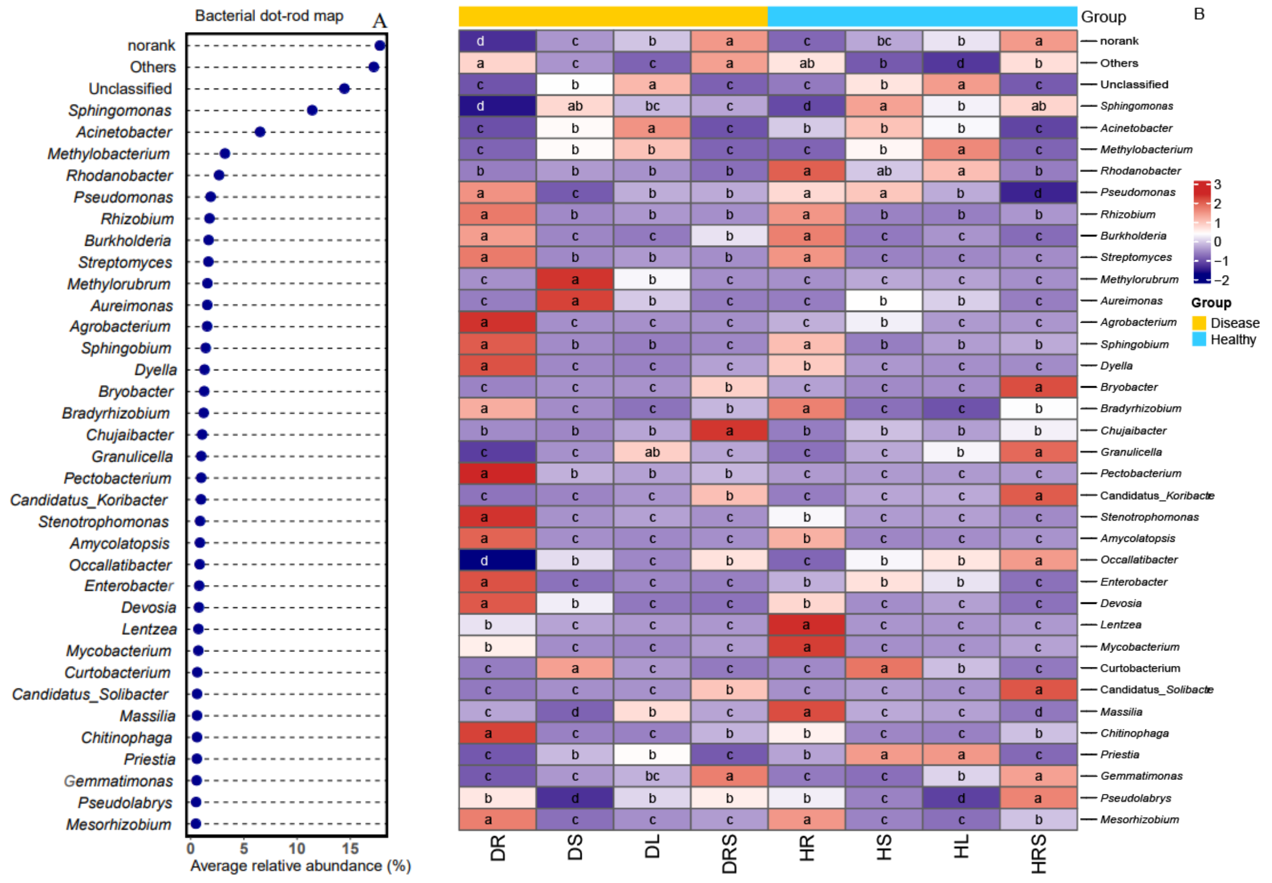

Supplementary Figure 7 Dot-rod heatmap of bacterial communities at genus level from long line pepper (LLP) plant rhizosphere soil (RS) and vegetative organs in both health states. in dot-rod map (the left, **A**), the horizontal coordinate indicates the average relative abundances of bacterial genera, which gradually decrease from the top to the bottom. Normalization in row was conducted in heatmap (**B**), different lowercase letters express significant difference in same row at $P < 0.05$; same lowercase letters express no significant difference in same row at $P \geq 0.05$ (ANOVA and LSD test).

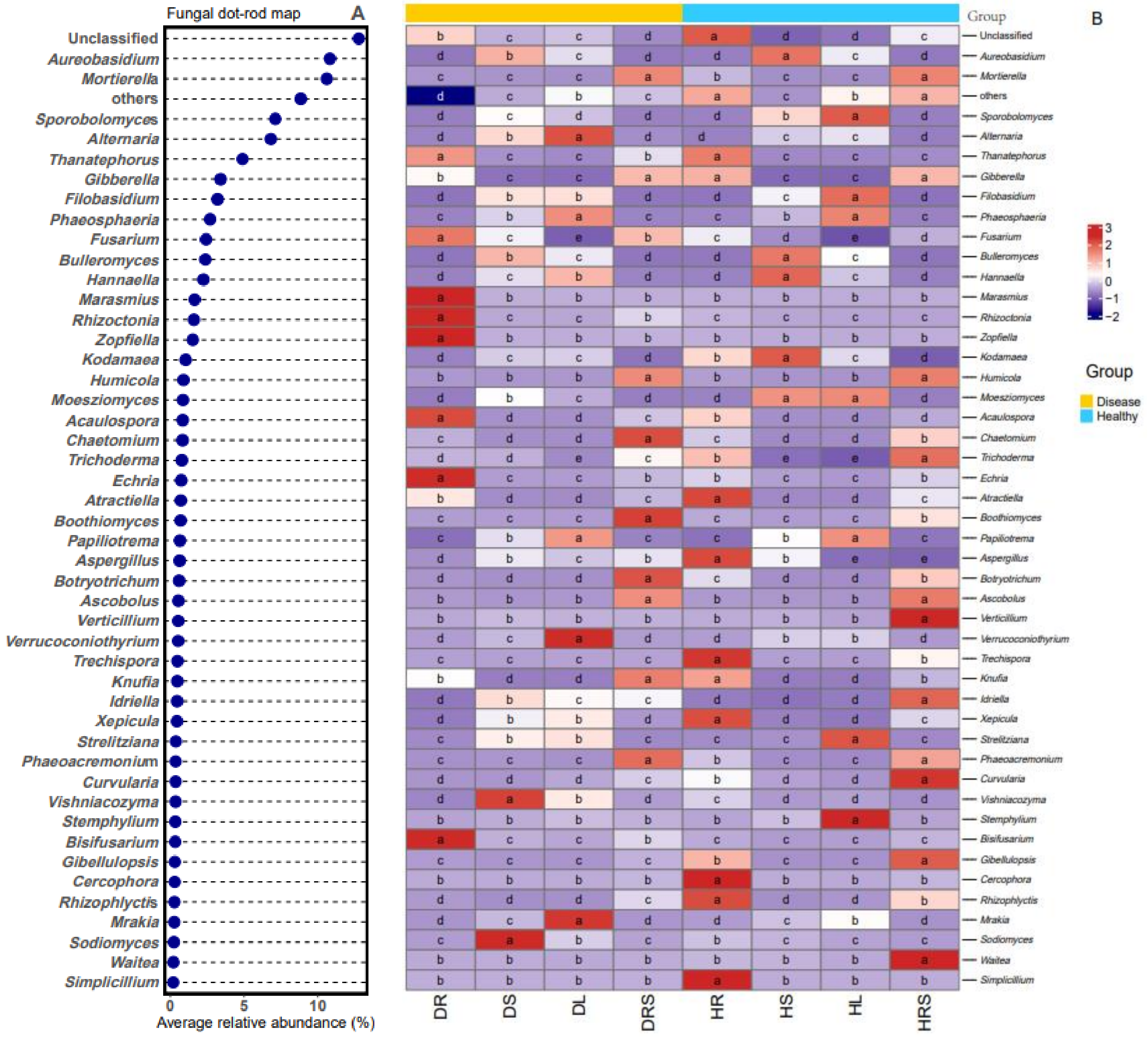

Unclassified
Aureobasidium
Mortierella
others
Sporobolomyces
Alternaria
Thanatephorus
Gibberella
Filobasidium
Phaeosphaeria
Fusarium
Bulleromyces
Hannaella
Marasmius
Rhizoctonia
Zopfiella
Kodamaea
Humicola
Moesziomyces
Acaulospora
Chaetomium
Trichoderma
Echria
Atractiella
Boothiomyces
Papiliotrema
Aspergillus
Botryotrichum
Ascobolus
Verticillium
Verrucoconiothyrium
Trechispora
Knufia
Idriella
Xepicula
Strelitziana
Phaeoacremonium
Curvularia
Vishniacozyma
Stemphylium
Bisifusarium
Gibellulopsis
Cercophora
Rhizophlyctis
Mrakia
Sodiomyces
Waitea
Simplicillium

Supplementary Figure 8 Dot-rod heatmap of fungal communities at the genus level from rhizosphere soil (RS) and vegetative organs of long line pepper (LLP) in two health states. In dot-rod map (the left, **A**), the horizontal coordinate indicates the average relative abundances of fungal genera, which gradually decrease from the top to the bottom. Normalization in row was conducted in heatmap (right, **B**), different lowercase letters express significant difference in same row at $P < 0.05$; same lowercase letters express no significant difference in same row at $P \geq 0.05$ (ANOVA and LSD test).

Correlation heatmap with Pearson coefficient (*r*) value between LLP agronomic performances, soil property parameters and the microbial α-diversity, relative abundances of leaf bacterial communities at the genus level

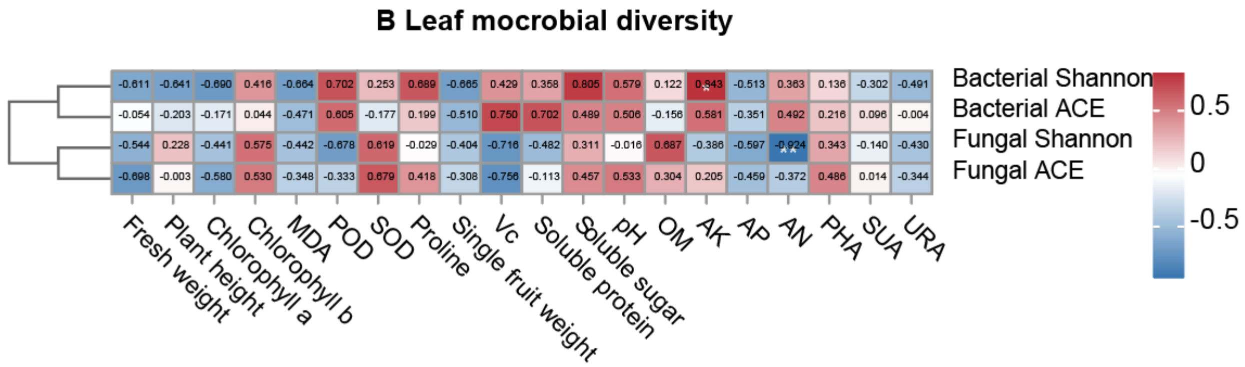

Supplementary Figure 9 Correlations heatmap of long line pepper (LLP) agronomic performances, soil property parameters and the microbial α-diversity (bacterial and fungal ACE and Shannon indexes). 6 repetitions of two health states were used to calculate the Pearson coefficient (*r*). No significance $P \geq 0.05$ was not labeled with *; significance $P < 0.001$ was labeled with ***, $P < 0.01$ **, $P < 0.05$ *. Normalization and classification were conducted in row. The following Supplementary Figures 10 and 11 are same.

Supplementary Figure 10 Correlations heatmap of LLP agronomic performances, soil property parameters and the relative abundances of leaf bacterial communities at the genus level. It was seen in the single PDF file Supplementary Figure 10.

Supplementary Figure 11 Correlations heatmap of LLP agronomic performances, soil property parameters and the relative abundances of leaf fungal communities at the genus level. It was seen in the single PDF file Supplementary Figure 11.

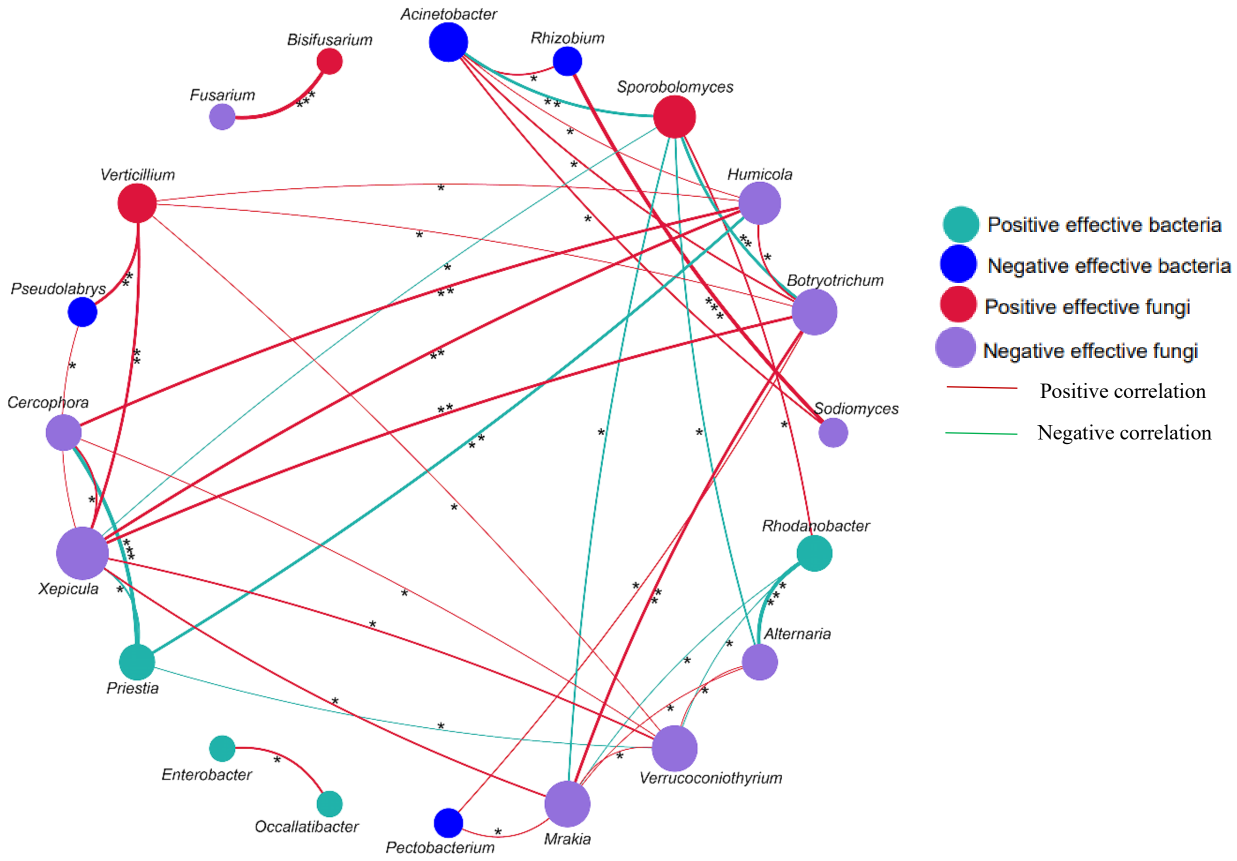

Supplementary Figure 12 The interactions among positive and negative effective microbes on agronomic traits of LLP plants (n = 6). Positive effective bacteria or fungi were the bacteria or fungi which exhibited significant positive correlations with some agronomic performances of LLP plants; negative effective bacteria or fungi were the bacteria or fungi which exhibited significant negative correlations with some agronomic performances of LLP plants. Significant correlation between two microbes $P < 0.001$ was labeled with ***, $P < 0.01$ **, $P < 0.05$ *. The thickness of the line between two microbes represents the absolute value of Pearson correlation coefficient. The co-occurrence network was completed using the Wekemo Bioincloud tool (https://www.bioincloud.tech) according to the reference (Gao, Y., Zhang, G., Jiang, S., Liu, Y. (2024). Wekemo Bioincloud: A user-friendly platform for meta-omics data analyses. *iMeta* 3: e175. https://doi.org/10.1002/imt2.175).

Regression analysis between specific microbial abundance profiles from LLP leaves and agronomic performance reductions in LLP plants

Supplementary Table 17 Regression equation of specific bacterial abundance profiles from LLP leaves and agronomic performance reductions in LLP plants (n = 6).

Bacteria (x)	plant indexes (y)	Regression equation	R^2	*P*
Acinetobacter	Fresh weight	y = -33.321x + 792.324	0.356	0.45
	Plant height	y = -1.6939x + 69.552	0.681	0.000535
	Chlorophyll a	y = -0.067x + 2.146	0.454	0.009039
	Chlorophyll b	y = 0.002x + 0.286	0.277	0.000158
	Vc	y = 0.0079x + 1.440	0.019	0.7
	Soluble protein	y = -0.000498x + 0.080	0.050	0.004
	Soluble sugar	y = 17.179x +333.8570	0.243	0.146
Mesorhizobium	Fresh weight	y = -1287.456x + 533.01	0.423	0.01
	Plant height	y = 0.475x + 50.789	0.000046	0.000762
	Chlorophyll a	y = -1.828x + 1.549	0.266	0.003
	Chlorophyll b	y = 0.030x + 0.307	0.045	0.000009
	Vc	y = -8.251x + 3.115	0.165	0.098
	Soluble protein	y = -0.007x + 0.075	0.08	0.000263
	Soluble sugar	y = 690.465x +4644.933	0.313	0.004
Pectobacterium	Fresh weight	y = -1605.616x +511.284	0.331	0.014
	Plant height	y = -60.667x + 54.590	0.374	0.000209
	Chlorophyll a	y = -3.471x +1.591	0.482	0.0012303
	Chlorophyll b	y = 0.172x +0.299	0.751	0.00000607
	Vc	y = 1.65x + 2.236	0.003	0.221
	Soluble protein	y = -0.046x + 0.077	0.171	0.000148
	Soluble sugar	y =848.074x +477.438	0.238	0.004
Pseudomonas	Fresh weight	y = -1096.971x +741.439	0.594	0.009
	Plant height	y = -1.289x + 51.221	0.001	0.004
	Chlorophyll a	y = -1.818x +1.923	0.509	0.003
	Chlorophyll b	y = 0.080x +0.286	0.627	0.000012
	Vc	y = -8.736x + 4.963	0.358	0.071
	Soluble protein	y = -0.004x + 0.075	0.005	0.002
	Soluble sugar	y =342.295x +360.672	0.404	0.038
Rhizobium	Fresh weight	y = -824.183x +793.539	0.631	0.008
	Plant height	y = -33.638x + 66.411	0.830	0.000074
	Chlorophyll a	y = -1.565x +2.101	0.708	0.0013
	Chlorophyll b	y = 0.042x +0.291	0.318	0.000052
	Vc	y =1.469x + 1.658	0.019	0.578
	Soluble protein	y = -0.021x +0.084	0.257	0.000839
	Soluble sugar	y = 420.971x +335.063	0.423	0.059

Note: Regression analysis was completed applying SPSS software, x represents the relative abundance of a specific microbe, y represents an index of agronomy.

Supplementary Table 18 Regression equation between specific fungal abundance profiles and agronomic performance reductions in LLP plants (n = 6).

Fungi (x)	plant indexes (y)	Regression equation	R^2	P
Alternaria	Fresh weight	y = -11.964x + 603.837	0.354	0.021
	Plant height	y = -597x + 60.419	0.698	0.000108
	Chlorophyll a	y = -0.025x + 1.771	0.467	0.003
	Chlorophyll b	y = 0.001x + 0.301	0.166	0.000022
	Vc	y =0.080x + 1.050	0.151	0.613
	Soluble protein	y = 0.00006199x + 0.073	0.006	0.00829
	Soluble sugar	y = 8.216x +398.185	0.430	0.014
Botryotrichum	Fresh weight	y = -9034.598x + 728.257	0.625	0.007
	Plant height	y = -201.085x +57.875	0.245	0.001343
	Chlorophyll a	y = -16.744x + 1.963	0.669	0.0126
	Chlorophyll b	y = 0.703x + 0.285	0.749	0.000004
	Vc	y =-34.305x +3.539	0.086	0.2
	Soluble protein	y = -1.35x + 0.079	0.088	0.000921
	Soluble sugar	y = 4445.058x +374.311	0.390	0.031
Cercophora	Fresh weight	y = -26080.908x + 805.139	0.782	0.02
	Plant height	y = -418.816x +57.148	0.160	0.002
	Chlorophyll a	y = -42.657x +2.020	0.652	0.002
	Chlorophyll b	y = 1.396x + 0.289	0.444	0.000028
	Vc	y =-171.266x +4.921	0.320	0.84
	Soluble protein	y = -0.588x + 0.083	0.250	0.000703
	Soluble sugar	y = 10708.691x +368.536	0.340	0.046
Humicola	Fresh weight	y = -7638.695x + 800.853	0.752	0.003
	Plant height	y = -145.022x +58.218	0.214	0.002
	Chlorophyll a	y = -13.122x +2.045	0.691	0.00131
	Chlorophyll b	y = 0.496x + 0.285	0.628	0.000014
	Vc	y =-46.198x +4.691	0.261	0.106
	Soluble protein	y = -0.185x + 0.083	0.277	0.000656
	Soluble sugar	y = 3144.110x +369.904	0.328	0.047
Mrakia	Fresh weight	y = -418.113x + 775.331	0.828	0.001343
	Plant height	y = -6.679x +56.639	0.167	0.002
	Chlorophyll a	y = -0.769x +2.045	0.873	0.000166
	Chlorophyll b	y = 0.031x + 0.283	0.893	8.263E-07
	Vc	y =-0.937x +3.153	0.040	0.253
	Soluble protein	y = -0.004x + 0.077	0.044	0.001077
	Soluble sugar	y = 245.534x +316.569	0.735	0.014
Sodiomyces	Fresh weight	y = -1591.803x + 623.585	0.407	0.017
	Plant height	y = -81.156x +61.628	0.836	0.000032
	Chlorophyll a	y = -3.074x +1.785	0.473	0.003
	Chlorophyll b	y = 0.047x + 0.304	0.070	0.000028
	Vc	y =8.970x +1.145	0.123	0.595
	Soluble protein	y = -0.010x + 0.075	0.010	0.000708
	Soluble sugar	y = 966.830x +401.418	0.386	0.016

Supplementary continued Table 18

Fungi	plant indexes	Regression equation	R^2	*P*
Verrucoconiothyrium	Fresh weight	y = -186.179x + 730.511	0.948	0.0001
	Plant height	y = -4.394x +58.354	0.417	0.000467
	Chlorophyll a	y = -0.331x +1.942	0.932	0.000035
	Chlorophyll b	y = 0.010x +0.293	0.512	0.000009
	Vc	y = -0.236x +2.742	0.014	0.259
	Soluble protein	y = -0.003x + 0.079	0.141	0.000479
	Soluble sugar	y = 101.385x +356.492	0.723	0.006
Xepicula	Fresh weight	y = -525.732x + 610.252	0.768	0.002
	Plant height	y = -8.698x +54.116	0.166	0.000571
	Chlorophyll a	y = -0.911x +1.720	0.718	0.000416
	Chlorophyll b	y = 0.033x +0.297	0.603	0.000002
	Vc	y =-2.145x +3.148	0.121	0.122
	Soluble protein	y = -0.014x + 0.079	0.361	0.000119
	Soluble sugar	y = 249.554x +435.842	0.445	0.005

Supplementary Table 19 Regression equation between relative abundance of root fungus *Fusarium* and agronomic performance reductions in LLP plants (n = 6).

fungus	plant indexes	Regression equation	R^2	*P*
Fusarium	Fresh weight	y = -31.986x+547.606	0.164	0.045
	Plant height	y = -0.683x+53.730	0.590	0.002
	Chlorophyll a	y = -0.058x+1.621	0.166	0.009
	Chlorophyll b	y = 0.004x+0.294	0.448	0.000012
	Vc	y = -0.412x+4.088	0.258	0.09
	Soluble protein	y = -0.003x+0.086	0.849	0.000012
	Soluble sugar	y = 1.499x+523.650	0.001	0.016
