# Supplementary material for: Microbial niche differentiation and agronomic performance of diseased Capsicum annuum
Source: Front Microbiol. 2025 Sep 3;16:1576486. doi: 10.3389/fmicb.2025.1576486 (PMC12440944; doi:10.3389/fmicb.2025.1576486)
Supplement: Supplementary file 2 [file Data_Sheet_2.pdf]

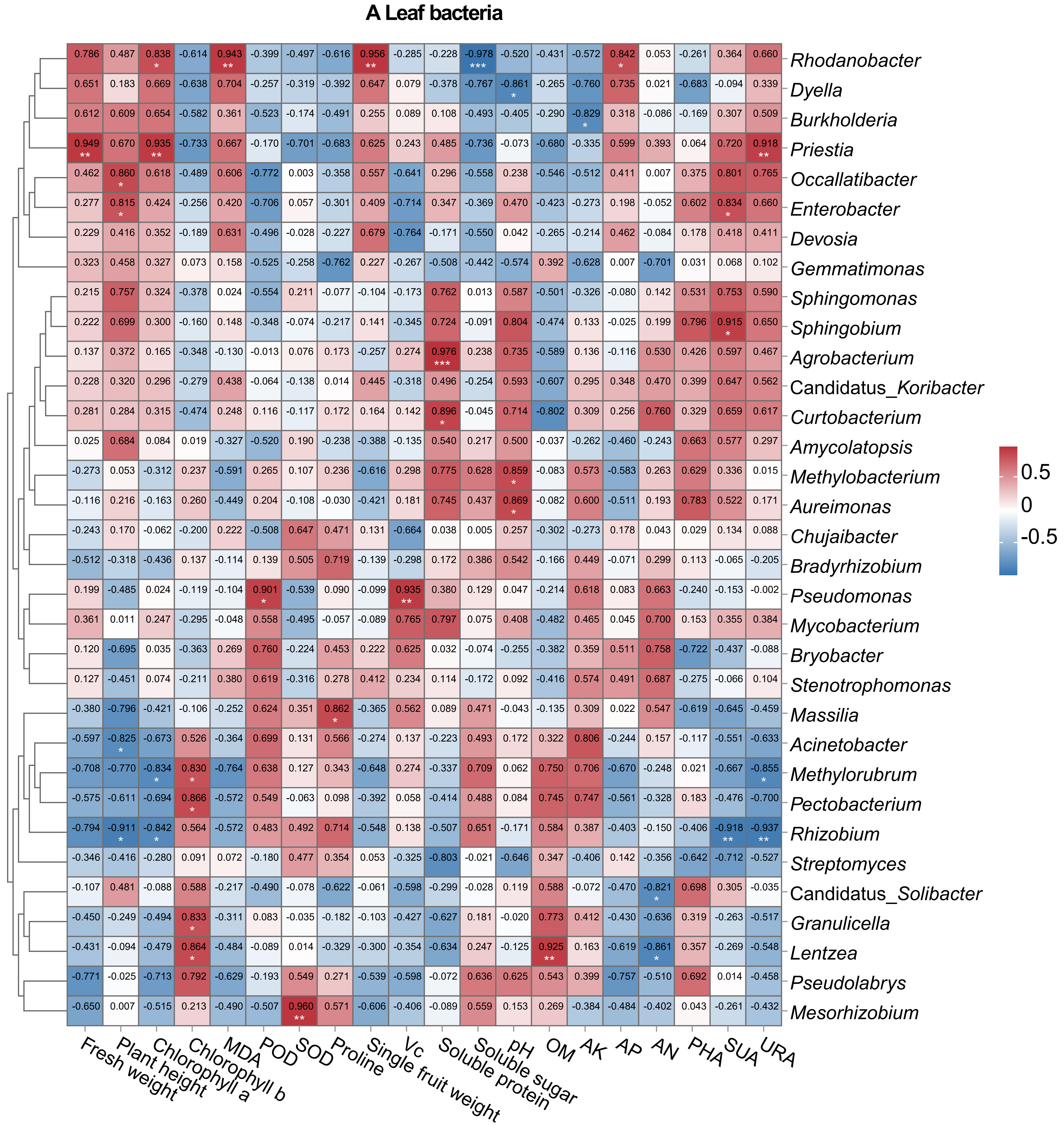

Supplementary Figure 10 Correlations heatmap of long line pepper (LLP) agronomic performances, soil property parametersand the relative abundances of leaf bacterial communities at the genus level.
