# Supplementary material for: Microbial niche differentiation and agronomic performance of diseased Capsicum annuum
Source: Front Microbiol. 2025 Sep 3;16:1576486. doi: 10.3389/fmicb.2025.1576486 (PMC12440944; doi:10.3389/fmicb.2025.1576486)
Supplement: Supplementary file 3 [file Data_Sheet_3.pdf]

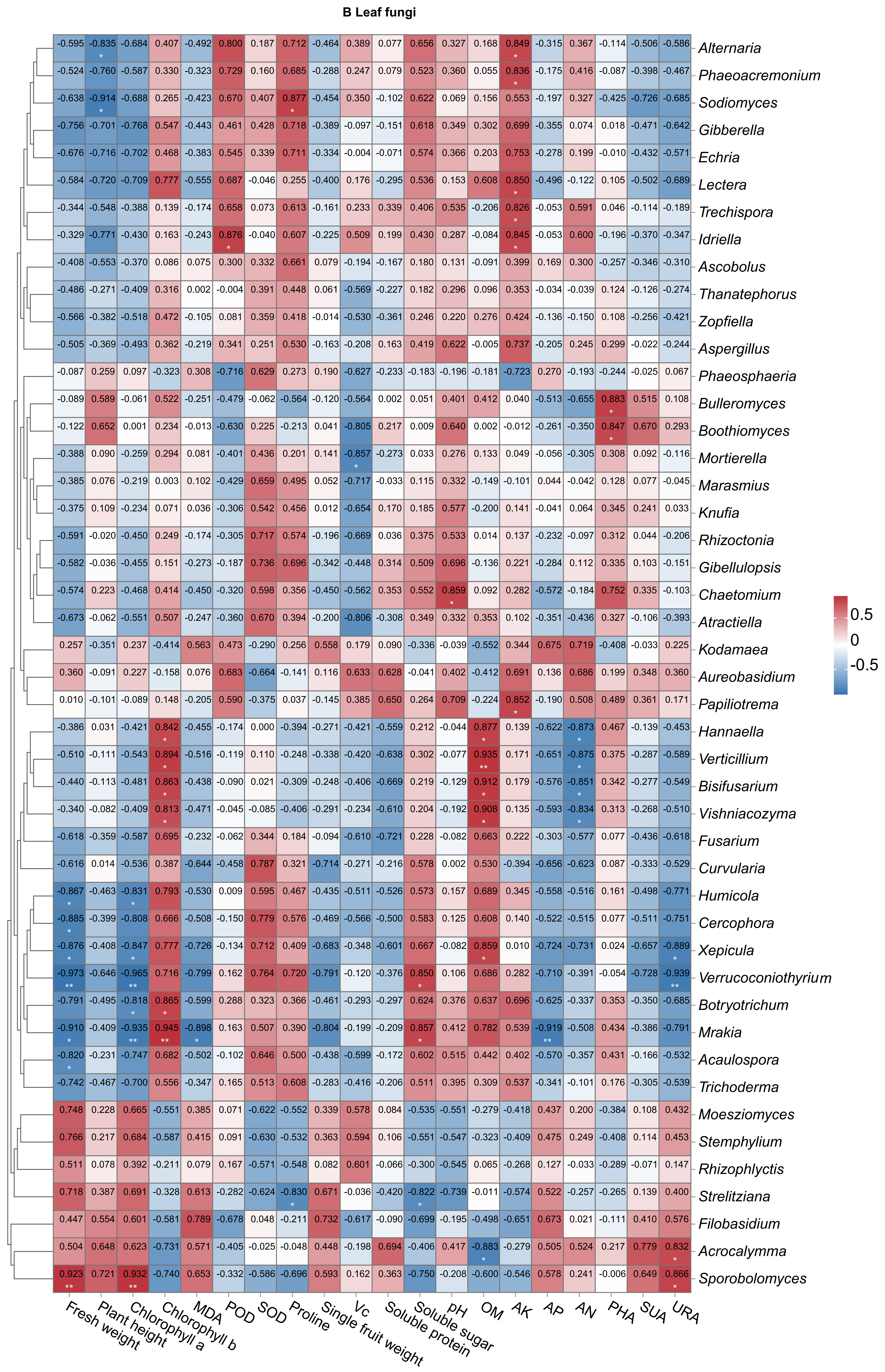

Supplementary Figure 11 Correlations heatmap of long line pepper (LLP) agronomic performances, soil property parameters and the relativeabundances of leaf fungal communities at the genus level.
